# LIX1 regulates YAP1 activity and controls the proliferation and differentiation of stomach mesenchymal progenitors

**DOI:** 10.1186/s12915-016-0257-2

**Published:** 2016-04-28

**Authors:** Jennifer McKey, Delphine Martire, Pascal de Santa Barbara, Sandrine Faure

**Affiliations:** PhyMedExp, INSERM U1046, CNRS UMR 9214, University of Montpellier, 34295 Montpellier, France

**Keywords:** Gastrointestinal tract, Mesenchymal progenitors, Smooth muscle cells, LIX1, YAP1, FGF pathway, Density-dependent cell proliferation

## Abstract

**Background:**

Smooth muscle cell (SMC) plasticity maintains the balance between differentiated SMCs and proliferative mesenchymal progenitors, crucial for muscular tissue homeostasis. Studies on the development of mesenchymal progenitors into SMCs have proven useful in identifying molecular mechanisms involved in digestive musculature plasticity in physiological and pathological conditions.

**Results:**

Here, we show that *Limb Expression 1* (*LIX1*) molecularly defines the population of mesenchymal progenitors in the developing stomach. Using in vivo functional approaches in the chick embryo, we demonstrate that *LIX1* is a key regulator of stomach SMC development. We show that *LIX1* is required for stomach SMC determination to regulate the expression of the pro-proliferative gene *YAP1* and mesenchymal cell proliferation. However, as stomach development proceeds, sustained *LIX1* expression has a negative impact on further SMC differentiation and this is associated with a decrease in YAP1 activity.

**Conclusions:**

We demonstrate that expression of LIX1 must be tightly regulated to allow fine-tuning of the transcript levels and state of activation of the pro-proliferative transcriptional coactivator YAP1 to regulate proliferation rates of stomach mesenchymal progenitors and their differentiation. Our data highlight dual roles for LIX1 and YAP1 and provide new insights into the regulation of cell density-dependent proliferation, which is essential for the development and homeostasis of organs.

**Electronic supplementary material:**

The online version of this article (doi:10.1186/s12915-016-0257-2) contains supplementary material, which is available to authorized users.

## Background

The gastrointestinal (GI) tract is a vital organ, highly conserved across vertebrate species and essential for the absorption of water and nutrients. During development, the GI tract arises from a primary uniform tube composed of mesoderm and endoderm. The mesoderm gives rise to the digestive mesenchyme, which in turn differentiates into multiple tissues, such as the submucosa and the musculature, which is composed of smooth muscle cells (SMCs) and interstitial cells of Cajal [1, 2]. The process of digestive mesenchyme development into SMCs is commonly decomposed into two major steps [3]. Mesenchymal progenitor cells first enter a determination program (that we will refer to as SMC determination), mainly characterized by the early expression of alpha smooth muscle actin (αSMA). Later during development, determined SMCs enter a more differentiated state (that we will refer to as SMC differentiation), mainly characterized by the expression of proteins involved in smooth muscle contractility, such as CALPONIN and CALDESMON.

Unlike many other mature cell types in the adult body, such as skeletal muscle cells, SMCs do not terminally differentiate but instead harbour a remarkable capacity to dedifferentiate. Indeed, SMCs have the unique ability to switch between a differentiated, quiescent contractile state and a highly proliferative and migratory phenotype in response to internal or external cues [1, 4]. SMC plasticity plays crucial roles in maintaining muscular tissue homeostasis during perinatal development and postnatal stages. In humans, the disruption of this balance is a major underlying cause of disease [4, 5]. Because tissue plasticity involves the reactivation of developmental processes, developmental studies of the process regulating the differentiation of mesenchymal progenitors into SMCs have proven to be useful in identifying the molecular mechanisms involved in the regulation of digestive musculature plasticity during normal development and in pathological conditions [6, 7].

Using a microarray approach to identify candidate genes in stomach mesenchyme development [8], an approach that had already enabled our group to characterize the RNA-binding protein RBPMS2 as a regulator of SMC differentiation and plasticity [6, 9], we screened for genes that demonstrated higher expression at the earliest stages of stomach development. This allowed us to identify *Limb Expression 1* (*LIX1*), a gene coding for a 281-amino acid protein. Although predictive in silico studies have shown that LIX1 has a double-stranded RNA binding domain, suggesting that it could be involved in RNA processing [10], no cellular function of LIX1 has yet been described. Chicken (*Gallus gallus*) *LIX1*, first identified in a gene expression screen to identify new markers of limb development, was shown to be expressed in the anterior and posterior intestinal portals, the early structures that invaginate to give rise to the primary intestinal tube [11]. Moreover, the arthropod homolog of *LIX1*, *lowfat*, has been characterized, through its interaction with the atypical cadherins *fat* and *dachsous*, as a component of the Hippo pathway [10, 12]. The Hippo pathway has been at the centre of many studies regarding its role in maintaining tissue homeostasis through the regulation of the balance between cell proliferation and differentiation. The key downstream regulator of the Hippo pathway is Yes-Associated Protein (YAP1), a transcriptional co-activator that mainly interacts with transcription factors of the TEAD family, which are essential in mediating YAP-dependent gene expression [13–15]. Indeed, the Hippo core cascade of kinases is activated when proper cell density and organ size are reached, leading, in humans, to the inhibitory phosphorylation of YAP1 on Serine-127 [16, 17]. This leads to decreased transcription of YAP1 mitogenic targets, resulting in a decrease in cell proliferation and entry into a more differentiated state [17]. Although LIX1 has recently been shown to stimulate progenitor proliferation during skeletal muscle regeneration in mouse [18], there is no evidence to date to support a role for LIX1 in regulating the activity of YAP1 in vertebrates.

In the present study, we investigated the roles of LIX1 and YAP1 during digestive SMC development. We show that *LIX1* is a novel and, thus far, unique marker of stomach mesenchymal progenitors and that its expression is strong and highly dynamic during development. We demonstrate that LIX1 stimulates the expression of *YAP1* transcripts and YAP1 activity and that both LIX1 and YAP1 are key regulators of the development of stomach mesenchymal progenitors. Finally, we show that YAP1 activity is required for the regulation of the proliferation and differentiation of the stomach mesenchyme.

## Results

### LIX1 defines stomach mesenchymal progenitors

**Fig. 1:**
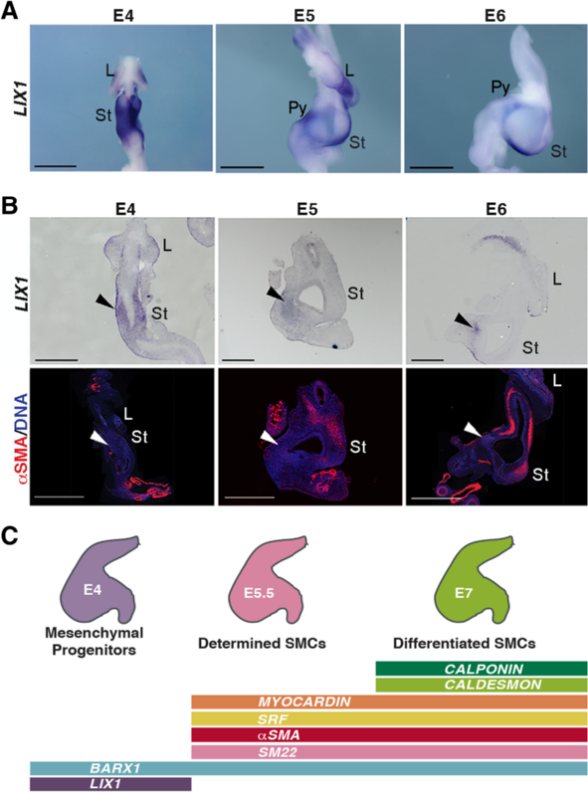
Transient expression pattern of *LIX1* in the developing chick stomach. **a** *LIX1* whole-mount in situ hybridization of embryonic day 4 (E4) to E6 stomachs. Scale bars, 1 mm. **b** Serial longitudinal sections of E4 to E6 stomachs analysed by in situ hybridization using the *LIX1* riboprobe and by immunofluorescence with anti-αSMA antibodies. Nuclei are visualized with Hoechst. Black arrowheads show the mesenchymal expression of *LIX1* at these stages. White arrowheads show the absence of αSMA in the *LIX1*-expressing domains. Scale bars, 500 μm. **c** Cartoon illustrating the steps of stomach mesenchyme development and summarizing Fig. 1a, b and Additional file 1: Figure S1A. L, Lung; St, Stomach; Pyl, Pylorus

We previously screened for genes that demonstrated higher expression at the earliest stages of stomach development [8] and found *LIX1* to be among them. Real-time quantitative PCR (RT-qPCR) analyses on stomach extracts confirmed the dynamic and transitory nature of *LIX1* expression during stomach development (Additional file 1: Figure S1A). While high levels of *LIX1* transcripts were detected at embryonic day 4 (E4), levels of *LIX1* transcripts quickly decreased with the onset of SMC determination (as visualized through the expression of *αSMA* and *SM22*), to finally barely detectable levels at E7, when SMC differentiation occurred (as shown by the high level of *CALPONIN* and *CALDESMON* expression; Additional file 1: Figure S1A). In parallel, we monitored the levels of *BARX1*, a marker of stomach mesenchyme [19], as well as *SRF* and its co-activator *MYOCARDIN*, which control SMC differentiation [20, 21], and found that, while the onset of *MYOCARDIN* expression occurs at E5.5, the stage of SMC determination, *SRF* and *BARX1* were detected throughout all examined stages. These results suggest that *LIX1* is an early marker of stomach development. We further studied the precise expression pattern of *LIX1* in the developing GI tract by in situ hybridization analysis (Additional file 1: Figure S1B). Strong *LIX1* expression was detected at E4 throughout the stomach mesenchyme and levels quickly decreased from E5 onwards (Fig. 1a, b). *LIX1* transcripts were mainly detected in the pylorus at E5 and in the most posterior part of the stomach at E6 (Fig. 1a, b). When comparing the dynamics of *LIX1* expression in the developing stomach with the kinetics of αSMA, the early marker of SMC determination in adjacent stomach sections, we observed that their expression domains appeared mutually exclusive (Fig. 1b). While *LIX1* expression was high in stomach mesenchymal progenitors, it progressively decreased with the onset of SMC determination, thus identifying *LIX1* as a novel and unique stomach marker, restricted to mesenchymal progenitors (Fig. 1c).

### *LIX1* silencing impairs mesenchyme determination and decreases YAP1 activity

**Fig. 2:**
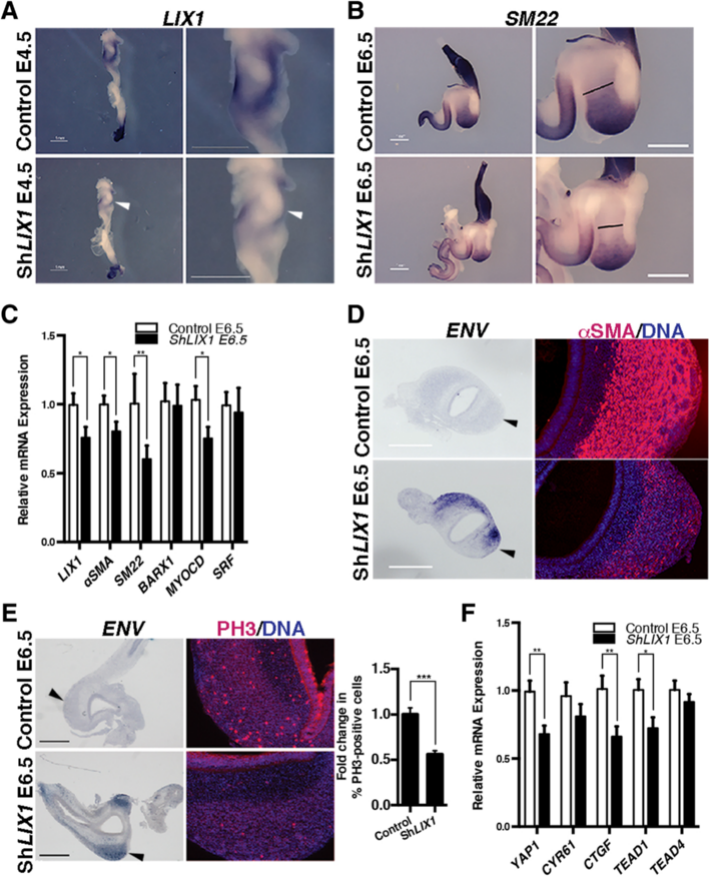
*LIX1* is required for mesenchymal progenitor proliferation and smooth muscle cell determination in the developing stomach. **a** *LIX1* whole-mount in situ hybridization of E4.5 gastrointestinal tracts. Scale bars, 1 mm. White arrowheads show the down-regulation of *LIX1* expression in Sh*LIX1*-expressing stomachs. **b** *SM22* whole-mount in situ hybridization of gastrointestinal tracts. Black bars locate the change in the *SM22*-expression domain. **c** RT-qPCR analysis of relative mRNA expression. Data were normalized to *GAPDH* expression. Normalized expression levels were converted to fold changes. Values are presented as the mean ± standard error of the mean (SEM) of *n* = 12 controls vs. *n* = 10 Sh*LIX1*-expressing stomachs. *P < 0.05; **P < 0.01 by one-tailed (*LIX1*, *αSMA* and *SM22*) or two-tailed (*BARX1*, *MYOCD* and *SRF*) Mann–Whitney tests. **d** Serial transverse sections of stomachs analysed either by in situ hybridization using the retroviral *Envelop* (*ENV*) riboprobe (scale bars, 500 μm) or by immunofluorescence with anti-αSMA antibodies. Nuclei were visualized with Hoechst. Black arrowheads in the *ENV* panels indicate the area that is imaged at high power in the αSMA panels. **e** Serial transverse sections of stomachs analysed either by in situ hybridization using the *ENV* riboprobe (scale bars, 500 μm) or by immunofluorescence using anti-PH3 antibodies. Nuclei are visualized with Hoechst. Black arrowheads in the *ENV* panels indicate the area imaged at high power in the PΗ3 panels. Graph represents the quantification of PH3-positive cells. Normalized expression levels were converted to fold changes. Values are presented as the mean ± SEM of *n* = 12 control vs. *n* = 10 Sh*LIX1*-expressing stomachs. ***P < 0.001 by two-tailed Mann–Whitney test. **f** RT-qPCR analysis of relative mRNA expression in control and Sh*LIX1*-expressing stomachs. Data were normalized to *GAPDH* expression. Normalized expression levels were converted to fold changes. Values are presented as the mean ± SEM of *n* = 12 controls vs. *n* = 10 Sh*LIX1*-expressing stomachs. *P < 0.05; **P < 0.01 by two-tailed Mann–Whitney tests

The complementarity between *LIX1* and αSMA expression prompted us to investigate whether *LIX1* is required for the process of stomach SMC determination. This was accomplished using the avian replication-competent retroviral (RCAS) transgenesis method that allows in vivo gain- or loss-of-function approaches of specific genes in the stomach mesenchyme (Additional file 2: Figure S2A) [6, 8, 19, 22]. We first performed *LIX1* loss-of-function experiments using RCAS(A)-Sh*LIX1* (short-hairpin RNA directed against *LIX1*) retroviruses. When injected into the presumptive domain of the developing stomach, RCAS(A)-Sh*LIX1* retroviruses led to a specific decrease in endogenous *LIX1* expression, demonstrated by in situ hybridization and RT-qPCR analyses (Fig. 2a, c). In situ hybridization analysis revealed a decrease in the expression of the SMC determination marker *SM22* in E6.5 Sh*LIX1*-expressing stomachs compared to controls (Fig. 2b) upon *LIX1* silencing. This was confirmed by RT-qPCR analysis (Fig. 2c). In contrast, injection of unrelated RCAS(A)-ShRNA retroviruses, which do not target *LIX1*, had no effect on *αSMA* expression (Additional file 3: Figure S3A). Moreover, when RCAS(A)-Sh*LIX1* retroviruses were co-injected with RCAS(B)-h*LIX1* retroviruses, which induce the expression of the human LIX1 protein insensitive to the chick-specific RCAS(A)-Sh*LIX1* retroviruses, normal expression of *αSMA* was restored, demonstrating the specificity of the Sh*LIX1* construct for *LIX1* (Additional file 3: Figure S3B). Levels of *BARX1* transcripts were comparable in Sh*LIX1*-expressing stomachs compared to controls, indicating that the patterning of the stomach was unaffected by *LIX1* silencing (Fig. 2c). We also observed a decrease in *MYOCARDIN* expression, while levels of *SRF* transcripts were not significantly affected in E6.5 Sh*LIX1*-expressing stomachs compared to controls (Fig. 2c). *LIX1* silencing induced a smaller determined-SMC territory, as demonstrated by in situ hybridization (Fig. 2b) and immunostaining analyses on Sh*LIX1*-expressing stomach sections compared to controls (Fig. 2d; Additional file 4: Table S1). The diminished expression of SMC determination markers was associated with a 40 % decrease in the rate of cell proliferation in Sh*LIX1*-expressing stomach sections compared to controls, as demonstrated by immunostaining analysis for phosphorylated histone 3-Ser10 (PH3; Fig. 2e), a standard marker of the G2/M transition [6]. These results are in line with a role for LIX1 in regulating cell proliferation, as previously shown in studies on cricket (*Gryllus bimaculatus*) and mouse that identified homologs of *LIX1* as positive regulators of cell proliferation [10, 18]. *Lowfat*, the arthropod homolog of *LIX1*, has been characterized, through its interaction with the atypical cadherins *fat* and *dachsous*, as a component of the Hippo pathway [10, 12]. As the key downstream regulator of the Hippo pathway is the pro-proliferative gene YAP1, we next investigated whether LIX1 regulates the expression of *YAP1* during this process. In situ hybridization and RT-qPCR analyses revealed that endogenous transcripts of *YAP1* and its transcriptional targets *CTGF* and *CYR61*, known to stimulate cell proliferation [15, 23], are abundant during early development of the stomach (E4–5.5; Additional file 5: Figure S4A,B). At this stage, their expression is detectable in both the mesenchymal and epithelial layers of the stomach, as demonstrated by RT-qPCR analyses on layer-dissociated stomach extracts (Additional file 5: Figure S4C). RT-qPCR analysis showed a reduction in the level of *YAP1* and its transcriptional targets *CTGF* and *CYR61* in Sh*LIX1*-expressing stomachs compared to controls (Fig. 2f). Although expression data were significant for *CTGF*, but not for *CYR61*, the results for both transcripts were consistent. We attribute the lack of significance for the second transcript to low statistical power rather than to absence of an effect. These results indicate that YAP1 activity was decreased in Sh*LIX1*-expressing stomachs compared to controls. Moreover, *LIX1* silencing also induced a decrease in the expression of the TEAD transcription factor *TEAD1* (Fig. 2f). Taken together, our results show that, when *LIX1* expression was silenced in the developing stomach, SMC determination was hindered. This was associated with a decrease in cell proliferation and a decrease in *YAP1* transcript levels and YAP1 activity in the developing mesenchyme. Our finding highlights the requirement of *LIX1* expression in the stomach mesenchymal progenitors to establish normal proliferation rates and allow proper SMC determination.

### LIX1 misexpression expands the determined SMC domain and stimulates cell proliferation and YAP1 activity

**Fig. 3:**
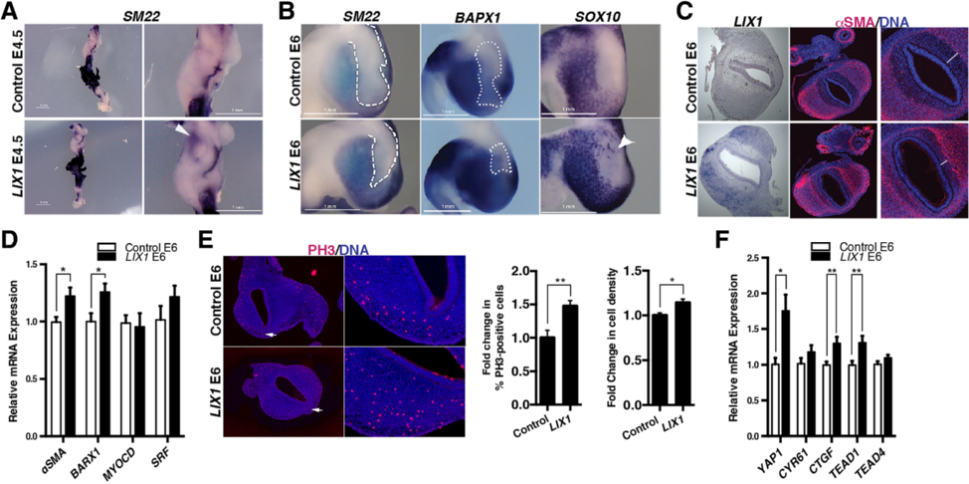
*LIX1* stimulates YAP1 activity, stomach mesenchymal progenitor proliferation and smooth muscle cell determination. **a** Whole-mount in situ hybridization of E4.5 gastrointestinal tracts using the *SM22* riboprobe. The white arrowhead indicates the premature expression of *SM22* in the *LIX1*-expressing stomach. **b** Whole-mount in situ hybridization of E6 stomachs using *SM22*, *BAPX1* and *SOX10* riboprobes. White dashed lines indicate the position of the intermuscular tendon in the stomach. The white arrowhead indicates the presence of ectopic *SOX10*-positive cells. Scale bars, 1 mm. **c** Serial transverse sections of E6 stomachs analysed either by in situ hybridization using the *LIX1* riboprobe or by immunofluorescence using anti-αSMA antibodies. Nuclei were visualized with Hoechst. White bars indicate the size of the submucosal domain in each condition. **d** RT-qPCR analysis of relative mRNA expression in E6 stomachs. Data were normalized to *GAPDH* expression. Normalized expression levels were converted to fold changes. Values are presented as the mean ± standard error of the mean (SEM) of *n* = 10 controls vs. *n* = 12 *LIX1*-expressing stomachs. *P < 0.05 by one-tailed (*αSMA*) or two-tailed (*BARX1*, *MYOCD* and *SRF*) Mann–Whitney tests. **e** Serial transverse sections of E6 stomachs analysed by immunofluorescence using anti-PH3 antibodies. Nuclei are visualized with Hoechst. White arrows in the left panels indicate the area imaged at high power in the PH3 panels. Graph represents the quantification of PH3-positive cells and cell density. Normalized expression levels were converted to fold changes. Values are presented as the mean ± SEM of *n* = 15 control vs. *n* = 15 *LIX1*-expressing stomachs. *P < 0.05; **P < 0.01 by two-tailed Mann–Whitney test. **f** RT-qPCR analysis of relative mRNA expression in E6 control or *LIX1*-expressing stomachs. Data were normalized to *GAPDH* expression. Normalized expression levels were converted to fold changes. Values are presented as the mean ± SEM of *n* = 10 controls vs. *n* = 12 *LIX1*-expressing stomachs. *P < 0.05; **P < 0.01 by one-tailed Mann–Whitney tests

We next induced a misexpression of *LIX1* in the stomach mesenchyme using RCAS(B)-*LIX1* retroviruses (Additional file 2: Figure S2A). This treatment did not drastically affect GI morphogenesis, as the morphology of *LIX1*-misexpressing stomachs resembled that of control embryos (Additional file 2: Figure S2B). We first observed a premature expression of SMC determination marker *SM22* as early as E4.5 in *LIX1*-misexpressing stomachs, whereas SMC determination had not yet taken place in controls, suggesting that *LIX1* misexpression facilitated SMC determination (Fig. 3a, white arrowhead). As a result, we observed at E6 that *LIX1*-misexpressing stomachs harboured an expanded determined SMC territory at the expense of the adjacent domains, mainly the intermuscular tendons and the submucosa. This was demonstrated both by whole-mount in situ hybridization, which showed a larger expression domain of determined SMC markers *SM22* and *BAPX1* [24] in *LIX1*-misexpressing stomachs compared to controls (Fig. 3b), and by αSMA immunostaining on sections, showing that sustained *LIX1* expression led to a decrease in the size of the submucosa (Fig. 3c, compare white bars). Accordingly, analysis of the enteric nervous system network using in situ hybridization of *SOX10* transcripts revealed that enteric nervous system precursors, which normally colonize the SMC domain specifically [8], had migrated into the adjacent tendon territory, further indicating an expanded SMC domain in *LIX1*-misexpressing stomachs compared to controls (Fig. 3b, white arrowhead). Further analysis by RT-qPCR confirmed that, compared to control stomachs, *LIX1*-misexpressing stomachs harboured higher levels of SMC determination marker *αSMA* and *BARX1* transcripts at E6, whereas *MYOCARDIN* and *SRF* levels were not significantly affected (Fig. 3d). Taken together, our in vivo results indicate that LIX1 is not only necessary for correct SMC determination, but that it also acts in favour of the process. These changes are associated with an increase in the rate of cell proliferation, as demonstrated by immunostaining analysis for PH3, and consequently to an increase in mesenchymal cell density in E6 *LIX1*-misexpressing stomachs compared to controls (Fig. 3e). The rate of cell death, however, was comparable in both conditions, as demonstrated by immunostaining analysis of cleaved CASPASE-3 (Additional file 6: Figure S5). Taking into account the positive effect of LIX1 on SMC proliferation and our previous results demonstrating that *LIX1* silencing impaired YAP1 expression and activity, we suspected that *LIX1* overexpression would stimulate the expression of genes in the YAP1 pathway. In fact, RT-qPCR analysis indicated a significant increase in the expression of *YAP1*, *CTGF* and *TEAD1*, and a slight increase in the expression of *CYR61* and *TEAD4* in *LIX1*-misexpressing stomachs compared to controls (Fig. 3f).

**Fig. 4:**
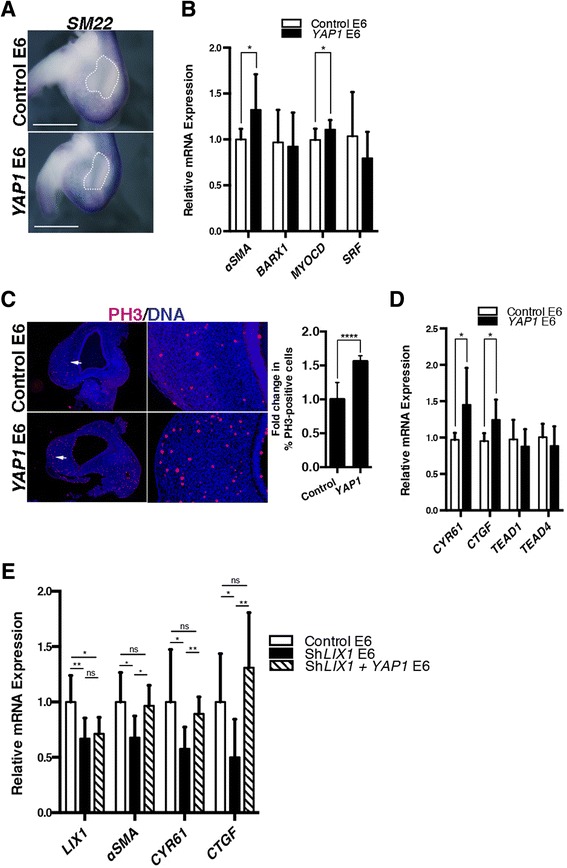
*YAP1* stimulates stomach mesenchymal progenitor proliferation and smooth muscle cell determination. **a** Whole-mount in situ hybridization of E6 stomachs using the *SM22* riboprobe. White dashed lines indicate the position of the intermuscular tendon in the stomach. Scale bars, 1 mm. **b** RT-qPCR analysis of relative mRNA expression in E6 stomachs. Data were normalized to *GAPDH* expression. Normalized expression levels were converted to fold changes. Values are presented as the mean ± standard derivation (SD) of *n* = 8 controls vs. *n* = 6 *YAP1*-expressing stomachs. *P < 0.05 by one-tailed Mann–Whitney tests. **c** Serial transverse sections of E6 stomachs analysed by immunofluorescence using anti-PH3 antibodies. Nuclei are visualized with Hoechst. White arrows in the left panels indicate the area imaged at high power in right panels. Graph represents the quantification of PH3-positive cells. Normalized expression levels were converted to fold changes. Values are presented as the mean ± standard error of the mean of *n* = 10 controls vs. *n* = 10 *YAP1*-expressing stomachs. ****P < 0.0001 by two-tailed Mann–Whitney test. **d** RT-qPCR analysis of relative mRNA expression in E6 stomachs. Data were normalized to *GAPDH* expression. Normalized expression levels were converted to fold changes. Values are presented as the mean ± SD of *n* = 8 controls vs. *n* = 6 *YAP1*-expressing stomachs. *P < 0.05 by one-tailed Mann–Whitney test. **e** RT-qPCR analysis of relative mRNA expression in E6 stomachs. Data were normalized to *GAPDH* expression. Normalized expression levels were converted to fold changes. Values are presented as the mean ± SD of *n* = 8 controls vs. *n* = 8 Sh*LIX1* vs. *n* = 6 Sh*LIX1* + *YAP1*-expressing stomachs. *P < 0.05; **P < 0.01 by one-tailed Mann–Whitney test. ns, not significant

The differences in YAP1 expression and activity observed in *LIX1*-misexpressing stomachs could be linked to the changes in the identity of the tissue associated with aberrant *LIX1* expression, or could be due to a role of YAP1 as a key relay in the establishment of the *LIX1* phenotype. We thus performed *YAP1* gain-of-function experiments in the developing stomach using RCAS(B)-*YAP1* retroviruses. We observed an expanded *SM22-*positive determined SMC domain in *YAP1*-misexpressing stomachs compared to control stomachs (Fig. 4a). RT-qPCR analysis indicated that *YAP1* misexpression did not affect the endogenous expression of *LIX1* (data not shown) and confirmed an increase in the transcript levels of the SMC determination markers *αSMA* and *MYOCARDIN* at E6 (Fig. 4b). Levels of *BARX1*, *SRF*, *TEAD1* and *TEAD4* were not significantly affected in *YAP1*-misexpressing stomachs compared to control stomachs (Fig. 4b, d). Moreover, changes in expression of SMC determination markers were associated with an increase in cell proliferation, as demonstrated by immunostaining analysis for PH3 (Fig. 4c). Our results thus demonstrate that LIX1 stimulates the endogenous level of *YAP1* transcripts and YAP1 activity and that sustained YAP1 activity phenocopies *LIX1* misexpression regarding stomach mesenchyme determination. Furthermore, when RCAS(A)-Sh*LIX1* retroviruses were co-injected with RCAS(B)-*YAP1* retroviruses, the expression of *LIX1* was not rescued (Fig. 4e). However, the restored YAP1 activity (monitored through the expression of *CYR61* and *CTGF* transcripts) rescued the expression of *αSMA* (Fig. 4e). Altogether, these data demonstrate that YAP1 is a key relay in the establishment of the *LIX1* phenotype.

### Endogenous *LIX1* expression is regulated by the FGF pathway during SMC determination

**Fig. 5:**
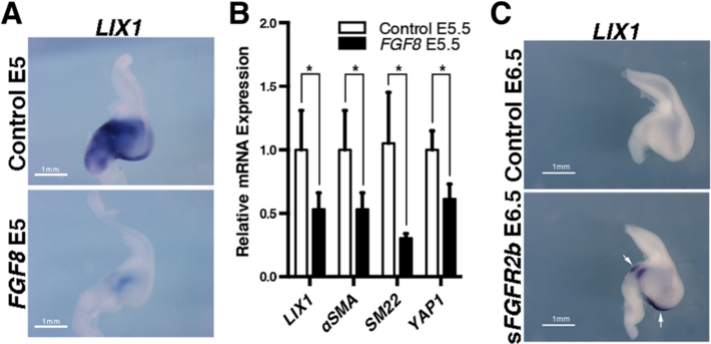
Endogenous *LIX1* expression is regulated by the FGF pathway during smooth muscle cell determination. **a** *LIX1* whole-mount in situ hybridization of E5 control *GFP*- and *FGF8-*expressing stomachs. **b** RT-qPCR analysis of relative mRNA expression in E5 stomachs. Data were normalized to *GAPDH* expression. Normalized expression levels were converted to fold changes. Values are presented as the mean ± standard deviation of *n* = 4 controls vs. *n* = 3 *FGF8*-expressing stomachs. *P < 0.05 by one-tailed Mann–Whitney test. Raw data are shown in Additional file 12. **c** *LIX1* whole-mount in situ hybridization of E6.5 control *GFP*- and s*FGFR2b-*expressing stomachs. Scale bars, 1 mm. White arrows show the domain of the s*FGFR2b*-expressing stomach in which the expression of *LIX1* is sustained

Collectively, our in vivo loss- and gain-of-function experiments demonstrate that *LIX1* expression must be finely regulated in the stomach mesenchyme to control the pool of progenitors required for correct SMC determination, presumably through the regulation of YAP1 activity. It has been shown that aberrant activation of the FGF pathway has a negative impact on stomach SMC determination [8]. As we report that *LIX1* silencing impaired SMC determination, we next investigated whether the expression of *LIX1* was under the control of the FGF signalling pathway. To address this question, we activated the FGF signalling pathway by misexpressing *FGF8* in the stomach mesenchyme using RCAS(A)-*FGF8* retroviruses and confirmed by RT-qPCR analysis that mesenchyme determination was hindered, as demonstrated by lower levels of *αSMA* and *SM22* transcripts in *FGF8-*misexpressing stomachs compared to controls (Fig. 5b). The upregulation of FGF activity was associated with a strong reduction in *LIX1* transcript levels compared to control stomachs, which was monitored by in situ hybridization (Fig. 5a) and confirmed by RT-qPCR analysis (Fig. 5b), and this was associated with a decrease in the levels of *YAP1* transcripts (Fig. 5b). These results suggest that sustained FGF activity during SMC determination phenocopies *LIX1* loss-of-function. Conversely, when using RCAS(B)-s*FGFR2b* retroviruses, which produce a secreted form of FGFR2b [8, 25], we found that inhibition of FGF pathway activity induced an increase in *LIX1* levels at E6.5 compared to control stomachs (Fig. 5c, white arrows). Taken together, these results suggest that the FGF pathway regulates the endogenous expression of *LIX1* and thereby maintains the proper levels necessary to ensure correct stomach mesenchyme determination.

### Sustained LIX1 expression decreases YAP1 activity and hinders SMC differentiation

**Fig. 6:**
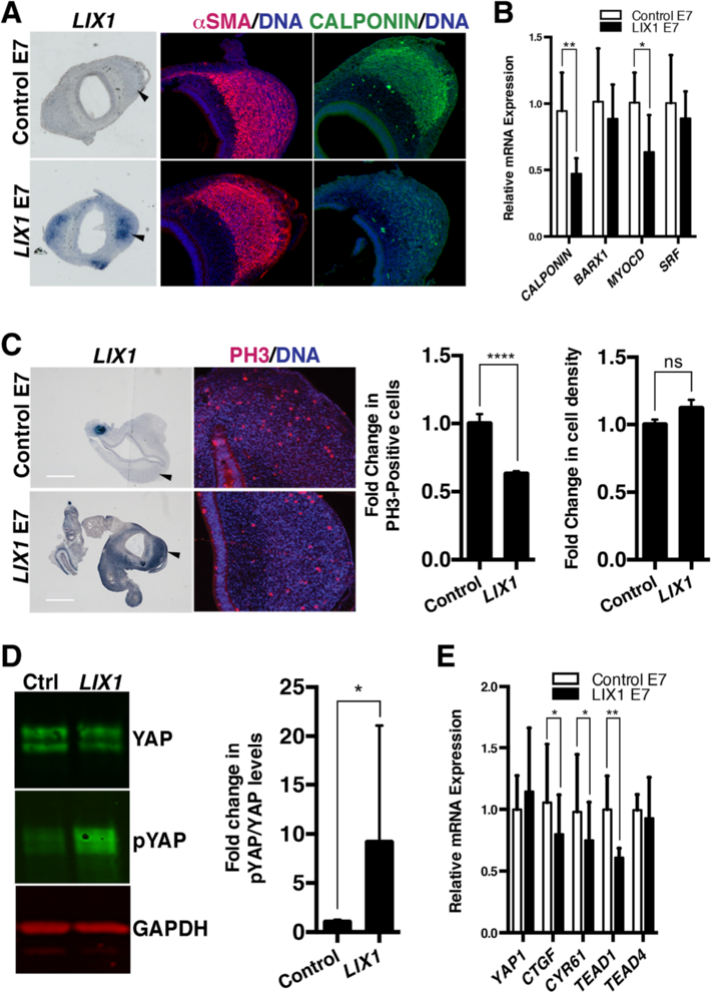
Sustained *LIX1* expression decreases YAP1 activity and hinders smooth muscle cell differentiation. **a** Serial transverse sections of E7 stomachs analysed either by in situ hybridization using the *LIX1* riboprobe or by immunofluorescence with anti-αSMA and anti-CALPONIN antibodies. Nuclei were visualized with Hoechst. Black arrowheads in the *LIX1* panels indicate the area imaged at high power in the αSMA and CALPONIN panels. **b** RT-qPCR analysis of relative mRNA expression in E7 stomachs. Data were normalized to *GAPDH* expression. Values are presented as the mean ± standard deviation (SD) of *n* = 7 controls vs. *n* = 6 *LIX1*-expressing stomachs. *P < 0.05; **P < 0.01 by one-tailed Mann–Whitney test. **c** Serial transverse sections of E7 stomachs analysed either by in situ hybridization using the *LIX1* riboprobe or by immunofluorescence with anti-PH3 antibodies. Black arrowheads in the *LIX1* panels indicate the area imaged at high power in the PH3 panels. Nuclei were visualized with Hoechst. Scale bars, 500 μm. Graphs represent the quantification of PH3-positive cells and cell density. Normalized expression levels were converted to fold changes. Values are presented as the mean ± standard error of the mean of *n* = 16 controls vs. *n* = 16 *LIX1*-expressing stomachs. ****P < 0.0001 by two-tailed Mann–Whitney tests. ns, not significant. **d** Western blot analysis of YAP and phospho-YAP (72 kDa) protein levels. Equal loading was verified by GAPDH expression (37 kDa). Graph represents the quantification of western blot data. Values are presented as the mean ± SD of *n* = 4 controls vs. *n* = 4 *LIX1*-expressing stomachs. *P < 0.05 by one-tailed Mann–Whitney test. Raw data are shown in Additional file 12. **e** RT-qPCR analysis of relative mRNA expression in E7 stomachs. Data were normalized to *GAPDH* expression. Normalized expression levels were converted to fold changes. Values are presented as the mean ± SD of *n* = 7 controls vs. *n* = 6 *LIX1*-expressing stomachs. *P < 0.05; **P < 0.01 by one-tailed Mann–Whitney tests

To further understand the role of *LIX1* in the development of the stomach mesenchyme, we next analysed the consequences of *LIX1* misexpression on SMC differentiation, the later step of SMC development. We found a reduction in the level of CALPONIN protein at E7 in *LIX1*-misexpressing stomachs, indicating that SMC differentiation was impaired (Fig. 6a). A strong reduction in *CALPONIN* transcript levels was also observed (Fig. 6b). Additionally, we observed a decrease in the expression of *MYOCARDIN*, while levels of *BARX1* and *SRF* transcripts were not significantly affected (Fig. 6b). The decrease in SMC differentiation markers in *LIX1*-misexpressing stomachs was also observed later in development at E8.5, suggesting that the reduced level of differentiation markers did not simply reflect a delay in stomach SMC development (Additional file 7: Figure S6). We found that *YAP1* misexpression also hindered CALPONIN expression, as demonstrated by immunostaining on stomach sections (Additional file 8: Figure S7A). This suggests that, while *LIX1* misexpression and YAP1 stimulation had a positive impact on SMC determination, they hindered SMC differentiation. Surprisingly, we found that, when *LIX1* expression was sustained in the developing stomach, the downregulation in the expression of SMC differentiation markers was associated with a lower rate of proliferation (Fig. 6c). Indeed, mesenchymal cell density was comparable in *LIX1*-misexpressing stomach compared to controls (Fig. 6c). It has been shown that the Hippo pathway acts as a sensor of cell density [16, 17], thus mediating the relationship between cell proliferation and cell contact inhibition of proliferation. As cell density becomes higher, the Hippo pathway is activated, resulting in an inhibitory phosphorylation of YAP1 and thus a decrease in cell proliferation [26]. Interestingly, we observed a decrease in YAP1 activity in *YAP1*-misexpressing stomachs at E7 by western blot analysis monitored through an increase of the inactive phosphorylated form of YAP1 compared to controls (Additional file 8: Figure S7B). The decrease in YAP1 activity was confirmed by RT-qPCR analysis, which showed lower transcript levels of *CTGF* in *YAP1*-misexpressing stomachs at E7 compared to controls (Additional file 8: Figure S7C). These results indicate that, while *YAP1* misexpression in the stomach stimulated YAP1 transcriptional activity at determination stages, a decrease in YAP1 activity was observed later on at differentiation stages. One possible explanation is that sustained *LIX1* expression led to a decrease in YAP1 activity consecutive to cell contact inhibition of proliferation, as a consequence of the early stimulation of mesenchymal progenitor proliferation, and this could be inhibitory for SMC differentiation. In line with this hypothesis, western blot analysis revealed an increase of the inactive phosphorylated form of YAP1 compared to controls (Fig. 6d). The decrease in YAP1 activity in *LIX1*-misexpressing stomachs at E7 was further confirmed by RT-qPCR analysis, which showed lower transcript levels of YAP1 targets *CYR61* and *CTGF* in *LIX1*-misexpressing stomachs compared to controls (Fig. 6e). Additionally, we observed a decrease in *TEAD1* transcript levels in *LIX1*-misexpressing stomachs compared to controls (Fig. 6e). These data indicate that Hippo signalling was activated as a result of sustained *LIX1* expression at E7. Altogether, our results demonstrate that *LIX1* has an early role in the process of stomach SMC determination, through the regulation of YAP1-induced mesenchymal progenitor proliferation. However, as stomach development proceeds, sustained *LIX1* expression has a negative impact on further SMC differentiation and this is associated with a decrease in YAP1 activity.

### The ability of LIX1 to regulate cell proliferation is dependent on cell density

**Fig. 7:**
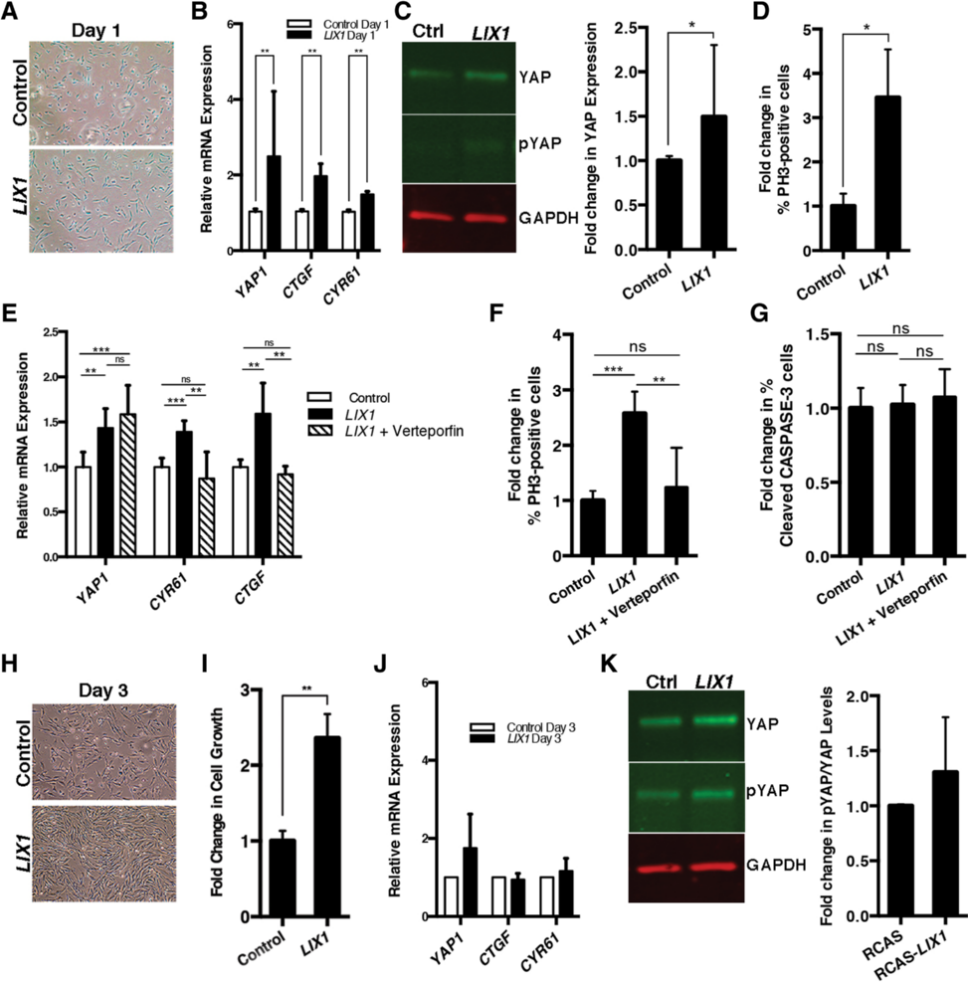
The ability of LIX1 to regulate cell proliferation is dependent on cell density DF-1 cells infected with control RCAS(A) or RCAS(B)-*LIX1* retroviruses. **a** Phase contrast photomicrograph at day 1. **b** RT-qPCR analysis of gene expression at day 1. Data were normalized to *UBIQUITIN* expression. Values presented are the mean ± standard deviation (SD) of *n* = 5 experiments. **c** Western blot analysis of YAP and phospho-YAP (72 kDa) protein levels. Equal loading was verified by GAPDH expression (37 kDa). Graph represents the quantification of western blot data. Values presented are the mean ± SD of *n* = 3 controls vs. *n* = 4 *LIX1*-expressing cell culture dishes. **d** Graphs represent the quantification of PH3-positive cells. A minimum of 1700 cells were analysed. Values presented are the mean ± standard error of the mean (SEM). **e** RT-qPCR analysis of gene expression. Values presented are the mean ± SD of six experiments*.* **f** Graphs represent the quantification of PH3-positive cells. A minimum of 1000 cells were analysed. Values presented are the mean ± SEM. **g** Graphs represent the quantification of cleaved CASPASE-3-positive cells. A minimum of 1000 cells were analysed. Values presented are the mean ± SEM. **h** Phase contrast photomicrograph of cells at day 3. **i** Quantification of cell growth at day 3. Values presented are the mean ± SEM of *n* = 10 experiments. **j** RT-qPCR analysis of gene expression. Values presented are the mean ± SD of *n* = 2 experiments. **k** Western blot analysis of YAP and phospho-YAP protein levels. Equal loading was verified by GAPDH expression. Graph represents the quantification of western blot data. Values presented are the mean ± SD of *n* = 2 experiments. Raw data for panels b, c, j and k are shown in Additional file 12. *P < 0.05; **P < 0.01; ***P < 0.001 (b, c, d, e, f and g: one-tailed Mann–Whitney test; i: two-tailed Mann–Whitney test). ns, non-significant

These results prompted us to investigate the role of *LIX1* in regulating both proliferation and contact inhibition of proliferation in heterologous cell cultures. DF-1 chicken fibroblasts were infected with empty RCAS(A) (control) or RCAS(B)-*LIX1* retroviruses and cultured for 5 days to ensure homogeneous expression. Then, cells were seeded at low density (Fig. 7a). As expected according to our in vivo results demonstrating a positive effect of LIX1 overexpression on the expression of YAP1 (Fig. 3f), after 1 day in culture (day 1) *LIX1*-expressing cells demonstrated a higher expression of *YAP1* transcript (Fig. 7b) and protein levels (Fig. 7c) compared to control cells. This greater expression was associated with higher transcript levels of YAP1 target genes *CTGF* and *CYR61* (Fig. 7b) and an increase in cell proliferation (Fig. 7d). Interestingly, when *LIX1*-expressing cells were treated with verteporfin, an inhibitor of the YAP-TEAD interaction that abrogates YAP activity but not its expression [27, 28], levels of *CTGF* and *CYR61* transcripts (Fig. 7e) and rates of proliferation (Fig. 7f) were comparable with those of control cells. Analysis of cell death in these cultures confirmed that this result was not due to a cytotoxic effect of verteporfin (Fig. 7g). These data demonstrate that, at low density, LIX1 regulates cell proliferation through modulation of YAP1 activity. After 3 days in culture, *LIX1-*expressing cells had grown faster than control cells (Fig. 7h, i). However, although *YAP1* expression in *LIX1*-expressing cells remained higher than in controls (Fig. 7j, k), the levels of *CTGF* and *CYR61* transcripts were similar to control levels. In addition, we observed an increase of the inactive phosphorylated form of YAP1 compared to controls in *LIX1*-expressing cells (Fig. 7k), indicating that YAP1 activity was downregulated at day 3 compared to day 1 (compare Fig. 7b with Fig. 7j). These data suggest that, under the influence of LIX1, a compensatory response to growing cell density took place. Indeed, while LIX1 acts to promote cell proliferation at low cell density, its pro-proliferation activity is abolished when cells had grown, suggesting that its ability to regulate cell proliferation is dependent upon cell density. In line with this hypothesis, when cells were seeded at high density, levels of *CTGF* and *CYR61* transcripts, YAP1 activity, and rates of proliferation were comparable in controls and *LIX1*-expressing cells after 1 day in culture (Additional file 9: Figure S8). The overexpression of LIX1 in vitro thus recapitulates the effects we had observed under misexpression of LIX1 in vivo during stomach mesenchyme development, suggesting that LIX1 drives an increase in cell density that feeds back on the system to block the activity of YAP1 and further proliferation.

## Discussion

Our study first identified *LIX1* as a novel and thus far unique marker of stomach mesenchymal progenitors. To our knowledge, *LIX1* is the first described gene to define the population of mesenchymal progenitors and to allow discrimination between undetermined and determined SMC states in the stomach. Collectively, our in vivo gain- and loss-of-function experiments clearly demonstrate that LIX1 is a key regulator of stomach mesenchyme development, by regulating both the determination and the differentiation of SMCs. Our study further demonstrates that YAP1 is a key relay of the function of LIX1 during these developmental processes.

We first identified LIX1 as an essential regulator of stomach mesenchyme determination. We thus suspect that the expression of *LIX1* must be tightly regulated in the developing mesenchyme to allow fine-tuning of the transcript levels and the state of activation of the pro-proliferative transcriptional coactivator YAP1, which in turn controls the rates of proliferation required for correct SMC determination. We further show that the FGF signalling pathway could be involved in the regulation of *LIX1* expression at determination stages. Most studies published so far have identified some regulators of YAP1 at the level of its activity, through its phosphorylation, localisation and stability [29]. Our study identifies LIX1 as a new regulator of *YAP1* mRNA levels, which is a novel finding. This could result from a regulation of the transcription of *YAP1* mRNA or from a regulation of its stability. Expression data were not always statistically significant for *TEAD4*. However, results were consistent between *TEAD1* and *TEAD* across all experiments. We thus attribute the lack of significance in some cases of effects on *TEAD4* to low statistical power rather than to absence of an effect. These functional in vivo data suggest that *LIX1* also regulates the expression of the TEAD transcription factors, which are essential in mediating YAP-dependent gene expression [15], indicating that LIX1 is an upstream regulator of YAP signalling. Further investigations will allow us to understand by which mechanisms LIX1 regulates the level of *YAP1* and *TEAD* transcripts. Interestingly, in silico studies have shown that LIX1 has a double-stranded RNA-binding domain, suggesting that it could be involved in mRNA or micro-RNA processing [10] and it has been shown that miR-506 and miR-375 regulate *YAP1* expression [30, 31]. It would thus be interesting to study whether LIX1 has a direct impact on *YAP1/TEAD* mRNA expression and/or stability.

We then demonstrated that LIX1 is an essential regulator of SMC differentiation. Intriguingly, while the pro-proliferative activity of LIX1 presumably facilitates SMC determination, LIX1 has a negative impact on further SMC differentiation. We suspect that high proliferative activity of LIX1 led to cell contact inhibition of proliferation, revealing the presence of a negative feedback loop on the endogenous expression and activity of YAP1 within the stomach mesenchyme to compensate for aberrant cell proliferation. Accordingly, we never observed hypertrophic stomachs under *LIX1* influence, suggesting that *LIX1* pro-proliferation activity is limited by the overall size of the stomach. In response to high cell density, the Hippo pathway regulates YAP1 activity through inhibitory phosphorylation [32] and we report here that the defect in SMC differentiation is associated with an increase in inactive phosphorylated YAP1 in *LIX1*-misexpressing stomachs. While the Hippo pathway has already been investigated in the context of gastrointestinal epithelia [28–30, 33], our study is the first to suggest a role for this pathway in regulating the proliferation and differentiation of the gastrointestinal mesenchyme. Along these lines, the next step would be to address the possible regulation of the Hippo pathway by *LIX1* in this developmental process. *Lowfat*, the arthropod homolog of *LIX1*, interacts with the atypical cadherins *fat* and *dachsous* and stabilizes FAT protein levels [12]. Although a recent study has shown that the vertebrate ortholog of FAT does not seem to regulate the Hippo pathway [34], FAT signalling has been shown to decrease YAP1 activity [35, 36]. One could thus speculate that, in the context of cell contact inhibition of proliferation, LIX1 participates more directly in the inhibition of YAP1 through the stabilization of FAT levels. Further investigations should focus on uncovering the potential molecular links that tie LIX1 to the regulation of YAP1 phosphorylation and transcriptional output.

Similarly to our conclusions for LIX1, we also report that while the pro-proliferative activity of YAP1 presumably facilitates SMC determination, it is sensitive to cell contact inhibition of proliferation and has a negative impact on further SMC differentiation. Because our misexpression experiments only led to mild overexpression of *YAP1* (ranging from 1.2- to 3-fold), we speculate that the native stomach mesenchyme is poised to respond to mild over-activity of YAP1 by turning on the negative feedback loop on YAP1 activity. This finding contrasts with those of previous studies where high levels of *YAP1* overexpression led to sustained proliferation and overgrowth of undifferentiated cells [17, 37]. In any case, the compensatory mechanisms resulting from *LIX1* or *YAP1* misexpression appeared to lock the determined mesenchyme in a state where the cells were neither proliferative nor differentiated. This state could simply reflect the requirement for a dynamic proliferation event between the determination and differentiation steps. By this hypothesis, because determined *LIX1/YAP1*-expressing cells are in contact inhibition of proliferation, differentiation could not be initiated. Alternatively, we could speculate that a certain level of YAP1 activity is necessary to initiate SMC differentiation, and because YAP1 activity has been turned off as a consequence of aberrant cell proliferation at the determination stage, differentiation could not be initiated. This second hypothesis highlights the possibility that YAP1 plays a dual role in regulating stomach mesenchyme progenitor development, both during the proliferative phase and later on during the differentiation phase. This hypothesis concords with emerging data showing that YAP1 regulates multiple signalling pathways, such as Wnt, BMP and Notch [38], and that Hippo signalling regulates Notch signalling [39]. Interestingly, all of these pathways are involved in the development of the GI tract [1, 6, 19, 40–42]. Further investigations are required to examine how YAP1 signalling is integrated in the regulation of SMC differentiation. YAP1 could be cooperating with two different transcription factors to regulate the processes of mesenchyme proliferation and SMC differentiation, similarly to what has recently been described during self-renewal of the intestinal epithelium [28]. In that system, the authors showed that YAP1 cooperates with Klf4 in promoting differentiation of intestinal goblet cells. Klf4 has been shown to abrogate the expression of myocardin, a major regulator of SMC differentiation [21], and of myocardin-induced expression of SMC genes [43], while YAP1 has been shown to interact with myocardin and interfere with its activity [13].

## Conclusion

Altogether, our results demonstrate that LIX1 is a novel and unique marker of digestive mesenchyme immaturity and a regulator of mesenchymal progenitor proliferation and differentiation through its capacity to regulate YAP1 activity and density-dependent proliferation. Additionally, we demonstrate that this activity of LIX1 is conserved in cell culture, suggesting that the mechanism of LIX1 action outlined here is not limited to the developing stomach mesenchyme. In light of these conclusions, it would be interesting to investigate whether the activity of *LIX1* is conserved throughout the more general context of organ size control and tissue regeneration. Finally, we have highlighted, through a developmental approach, three properties of *LIX1* that could make it essential in cancer research. LIX1 defines an immature state of stomach smooth muscle, regulates cell proliferation within this immature mesenchyme and regulates the activity of the oncogene YAP1. These three properties thus point to the interest of further studies to examine the possible function of *LIX1* in tumorigenesis and tumour progression.

## Methods

### Chick embryonic GI tissues

Fertilized White Leghorn eggs from the Haas Farm (France) were incubated at 38 °C in humidified incubators. Embryos were staged according to Hamburger and Hamilton [44]. Isolation of mesodermal and endodermal layers from stage 25 stomachs (referred to as E5) was performed as previously described [8]. The efficiency of dissections was evaluated by monitoring the expression of *SHH* and *BARX1*, which are specific markers of the epithelial and mesenchymal layers, respectively.

### Avian retroviral misexpression system and constructs

Chick *LIX1* full-length cDNA was isolated from total mRNA extracts of E5 stomachs. The mouse *YAP1*, the chick full-length *LIX1*, the human full-length *LIX1* and the Short hairpin RNA of *LIX1* (Gallus target sequence: TCT TTG CAG CTG GTG ATT G, referred to as Sh*LIX1*) associated with the mouse U6 promoter were cloned into the shuttle vector Slax13 and then subcloned into the Replication-Competent Avian Leucosis Sarcoma virus strain A (RCAS(A)) or strain B (RCAS(B)) vectors. Using RCAS vectors with two different envelopes (A and B) allows the introduction of two genes into a single cell [45]. *FGF8*, *sFGFR2b* and *GFP* retroviral constructs have been previously described [8]. RCAS(A)-sh*PROX1* retrovirus [46] served as unrelated RCAS-ShRNA retroviruses. Retroviral constructs were transfected into the chicken DF-1 fibroblast cell line (ATCC-LGC) to produce retroviruses. Retroviruses were titered using standard techniques and injected into the splanchnopleural mesoderm of E1.5 chicken embryos to target the stomach mesenchyme [22]. Embryos were co-injected with RCAS-*GFP* to allow screening of correctly targeted stomachs. Eggs were then placed at 38 °C until harvested. Efficient retroviral infection was confirmed by in situ hybridization analysis on paraffin sections using *ENV* probes or, in *LIX1* misexpression experiments only, *LIX1* probes. Infection with RCAS-*GFP* retroviruses does not affect chick stomach development (Additional file 10: Figure S9). Stomach phenotypes from infected embryos were analysed by comparison with uninfected control embryos incubated at the same time.

### Cell cultures and analysis

The chicken DF-1 fibroblast cell line was cultured as previously described [22]. Cell growth in DF-1 cultures was assessed using the Muse Count and Viability reagent following the manufacturer’s specifications (Muse Cell Analyzer – Millipore). DF-1 cells were plated on plastic at 2000 cells/cm^2^ to obtain low-density cultures and 6000 cells/cm^2^ to obtain high-density cultures. Verteporfin (Sellekchem) was used applied to DF-1 cells for 20 hours at a final concentration of 1 μM.

### In situ hybridization and immunofluorescence staining

Dissected GI tissues were fixed in 4 % paraformaldehyde at room temperature for 30 minutes, washed in PBS, gradually dehydrated in methanol and stored at −20 °C before processing for whole-mount in situ hybridization as previously described [8, 22]. For sections, GI tissues were fixed in 4 % paraformaldehyde at room temperature for 30 minutes, washed in PBS, gradually dehydrated in ethanol and embedded in paraffin. 10-μm sections were cut using a microtome and collected on poly-L-lysine-coated slides (Thermo Fisher). Partial chick *YAP1*, *CTGF* and *CYR61* cDNAs were isolated from total mRNA extracts of E5 stomachs. In situ hybridization experiments on whole-mount and paraffin sections were carried out as previously described [24] using chick *LIX1* and *YAP1* probes and published *SM22*, *BAPX1*, *SOX10* and *ENV* probes [8, 19, 24]. Immunofluorescence studies were performed on paraffin sections using polyclonal antibodies against aSMA (Abcam Cat# ab5694 RRID:AB_2223021 1:400 dilution), anti-Phospho-Histone H3-Ser10 (PH3) (Millipore Cat# 06–570 RRID:AB_310177, 1:300 dilution), cleaved CASPASE-3 (Cell Signaling Technology Cat# 9664S RRID:AB_331453, 1:400 dilution) and monoclonal antibodies against CALPONIN (Abnova Cat# MAB1512 RRID:AB_1672405, 1:500 dilution). Nuclei were labelled with Hoechst (Invitrogen). In vivo proliferation rates were assessed by counting the number of PH3-positive cells relative to the total number of nuclei in the section. Cell density was assessed on images of stomach sections by calculating the area occupied by Hoechst-stained nuclei relative to the total area of the section.

### Reverse transcription and quantitative polymerase chain reaction (RT-qPCR)

Total RNA was extracted from stomachs or cell cultures with the HighPure RNA Isolation kit (Roche). Reverse transcription was performed using the Verso cDNA synthesis kit (Thermo Scientific) and RT-qPCR was performed using LightCycler technology (Roche Diagnostics). PCR primers (Additional file 11: Table S2) were designed using the LightCycler Probe Design 2.0 software. Each sample was analysed in three independent experiments done in triplicate. Expression levels were determined with the LightCycler analysis software (version 3.5) relative to standard curves. Data were represented as the mean level of gene expression relative to the expression of the reference genes *UBIQUITIN* or *GAPDH*. Relative mRNA expression was calculated using the 2^–ΔΔCT^ method [47].

### Western blotting

DF-1 cells and chick stomachs were re-suspended in lysis buffer (20 mM Tris pH 8, 50 mM NaCl, 1 % NP40, cOmplete EDTA-free Protease Inhibitor Cocktail (Roche)); 10 μg of total protein lysates were boiled in SDS-PAGE sample buffer, separated by 10 % SDS-PAGE and transferred to nitrocellulose membranes. Membranes probed with rabbit anti-phospho-YAP (Ser127; Cell Signaling Technology Cat# 4911S RRID:AB_2218913, 1:1000 dilution), anti-YAP/TAZ (Cell Signaling Technology Cat# 8418S RRID:AB_10950494, 1:1000 dilution) or anti-GAPDH (Sigma-Aldrich Cat# G9545 RRID:AB_796208, 1:5000 dilution) antibodies overnight. Glyceraldehyde-3-phosphate dehydrogenase (GAPDH) expression was used to confirm equal loading. All immunoblots were developed and quantified using the Odyssey infrared imaging system (LICOR Biosystems) and infrared-labelled secondary antibodies.

### Statistical analysis

Data were analysed by performing two-tailed or, when appropriate, one-tailed Mann–Whitney tests using GraphPad Prism 6 software. Values of n represent the number of biological replicates. Each value used for statistical analyses is the mean of three technical replicates. Results were considered significant when *P* < 0.05 (*), *P* < 0.01 (**), *P* < 0.001 (***) or *P* < 0.0001 (****).

### Photography

Images were acquired using a Nikon Multizoom AZ100 stereomicroscope and a Carl Zeiss AxioImager microscope. Images presented in the figures are representative of the main phenotype observed in the population of infected embryos (Additional file 4: Table S1).

### Availability of data and materials

Data supporting the results of this article are available in Additional file 12.

## Additional files

Additional file 1: Figure S1. Comparison of *LIX1* expression with smooth muscle cell development markers. (A) RT- qPCR analysis of the endogenous relative mRNA expression of *LIX1*, *BARX1*, *SRF*, *aSMA*, *SM22*, *MYOCARDIN (MYOCD)*, *CALPONIN* and *CALDESMON* expression in E4, E5.5, E7 and E8.5 stomachs. (B) *LIX1* whole-mount in situ hybridization of E4 to E7 gastrointestinal tracts. Strong *LIX1* expression is detected in the stomach and colon as early as E4, decreasing over time to become no longer detectable from E7 onwards. We also noted a strong expression of *LIX1* in the associated lungs and anal plate. Scale bars, 1 mm. L, Lung; St, Stomach; C, Colon; AP, Anal plate. (JPG 427 kb) Additional file 2: Figure S2. RCAS retroviral infection. (A) Schematic representation of RCAS retroviral infection. RCAS(A)-Sh*LIX1* or RCAS(B)-*LIX1* retroviral particles are injected in the splanchnopleura, between somites 3 and 7 of embryos at E1.5. NT, Neural tube; NC, Notochord. (B) *LIX1* whole-mount in situ hybridization on E7.5 control or *LIX1*-expressing stomachs. (JPG 460 kb) Additional file 3: Figure S3. Specificity of the Sh*LIX1* construct for *LIX1* mRNA. (A) Analysis of *LIX1*, *αSMA* and *BARX1* expression by RT-qPCR in E6.5 control and RCAS-Sh*PROX1*-expressing stomachs. Data were normalized to *GAPDH* expression. Normalized expression levels were converted to fold changes. Values are presented as the mean ± standard error of the mean of *n* = 8 controls vs. *n* = 8 Sh*PROX1*-expressing stomachs. ns, Not significant by one-tailed (for *LIX1*) or two-tailed (for *αSMA* and *BARX1*) Mann–Whitney tests. (B) Analysis of *LIX1*, *αSMA* and *BARX1* expression by RT-qPCR in E6.5 control stomachs, RCAS(A)-Sh*LIX1*- expressing stomachs or RCAS(A)-Sh*LIX1*/RCAS(B)-h*LIX1*-co-expressing stomachs. Data were normalized to *GAPDH* expression. Normalized expression levels were converted to fold changes. Values are presented as the mean ± standard deviation of *n* = 12 controls vs. *n* = 8 Sh*LIX1* vs. *n* = 8 Sh*LIX1+* h*LIX1*-expressing stomachs. **P* < 0.05 by one-tailed Mann–Whitney test. ns, Not significant. (JPG 170 kb) Additional file 4: Table S1. Phenotype quantification. Quantification of embryos harbouring an abnormal stomach muscle phenotype, as demonstrated by in situ hybridization or immunostaining, following injection of RCAS(A)-Sh*LIX1* (*LIX1* loss-of-function) or RCAS(B)-*LIX1* (*LIX1* gain-of-function). (PDF 40 kb) Additional file 5: Figure S4. Expression pattern of *YAP1* and its transcriptional target genes during gastrointestinal development. (A) *YAP1*, *CTGF*, *CYR61* and *SM22* whole-mount in situ hybridization of E4–8 gastrointestinal tracts. L, Lung; St, Stomach; Co, Colon. (B) RT- qPCR analysis of the endogenous relative mRNA expression of *YAP1*, *CTGF*, *CYR61* in E4–8.5 stomachs. (C) RT-qPCR analysis of the endogenous relative mRNA expression of *YAP1*, *CYR61*, *CTGF*, *SHH*, *αSMA* and *BARX1* in mesenchymal and endodermal layers dissected from E5 stomachs. Total, Whole stomach; Epithelium, Epithelial layer; Mesenchyme, Mesenchymal layer. Values are presented as the mean ± standard deviation of *n* = 3 experiments. **P* < 0.05 by one-tailed Mann–Whitney test. ns, Not significant. Raw data for panel C are shown in Additional file 12. (JPG 1455 kb) Additional file 6: Figure S5. Sustained *LIX1* expression does not affect apoptosis. Transverse sections of E6 control and *LIX1*-expressing stomachs analysed by immunohistochemistry with anti-cleaved CASPASE-3 antibodies. Black arrows indicate the area imaged at high power in the cleaved-CASPASE-3 panels. Black arrowheads point to cleaved CASPASE-3-positive apoptotic cells. (JPG 402 kb) Additional file 7: Figure S6. Sustained *LIX1* expression hinders smooth muscle cell differentiation. Analysis of *CALPONIN* and *CALDESMON* expression by RT-qPCR in E8.5 control *GFP*- and *LIX1*-expressing stomachs. Data were normalized to *GAPDH* expression. Normalized expression levels were converted to fold changes. Values are presented as the mean ± standard deviation of *n* = 4 controls vs. *n* = 5 *LIX1*-expressing stomachs. ***P* < 0.01 by one-tailed Mann–Whitney test. ns, Not significant. Raw data are shown in Additional file 12. (JPG 91 kb) Additional file 8: Figure S7. Sustained *YAP1* expression hinders smooth muscle cell differentiation. (A) Serial transverse sections of E7 control *GFP*- and *YAP1*-expressing stomachs analysed either by in situ hybridization using the *ENV* riboprobe or by immunofluorescence with anti-αSMA and anti-CALPONIN antibodies. Nuclei were visualized with Hoechst. (B) Western blot analysis of YAP and phospho-YAP (72 kDa) levels in protein extracts from control *GFP*- and *YAP1*-expressing stomachs. Equal loading was verified by GAPDH expression (37 kDa). Graph represents the quantification of western blot data. Normalized expression levels were converted to fold changes. Values are presented as the mean ± standard deviation of *n* = 6 controls vs. *n* = 6 *YAP1*-expressing stomachs. **P* < 0.05 by one-tailed Mann–Whitney test. (C) RT-qPCR analysis of relative mRNA expression in E7 control *GFP*- and *YAP1*-expressing stomachs. Data were normalized to *GAPDH* expression. Normalized expression levels were converted to fold changes. Values are presented as the mean ± standard deviation of *n* = 8 controls vs. *n* = 6 *YAP1*-expressing stomachs. **P* < 0.05 by one-tailed Mann–Whitney test. (JPG 2004 kb) Additional file 9: Figure S8. The pro-proliferative effect of LIX1 is abolished when DF-1 cells are seeded at high density. (A) RT-qPCR analysis of relative mRNA expression in DF-1 cells infected with RCAS(B)-*GFP* control or RCAS(B)-*LIX1* plated at high density and harvested at day 1. Data were normalized to *GAPDH* expression. Normalized expression levels were converted to fold changes. Values are presented as the mean ± standard deviation (SD) of *n* = 2 experiments. Raw data for panel A are shown in Additional file 12. (B) Examination of proliferation in *GFP*-expressing cells (control) and *LIX1*-expressing cells. Graphs represent the quantification of PH3-positive cells. Normalized expression levels were converted to fold changes. Values are presented as the mean ± SD of *n* = 7 experiments. ns, Not significant by two-tailed Mann–Whitney test. (C) Western blot analysis of YAP and phospho-YAP (72 kDa) levels in protein extracts from *GFP-* and *LIX1*-expressing cells. Equal loading was verified by GAPDH expression (37 kDa). Graph represents the quantification of western blot data. Normalized expression levels were converted to fold changes. Values are presented as the mean ± SD of *n* = 7. (JPG 721 kb) Additional file 10: Figure S9. Infection with RCAS-*GFP* retroviruses does not affect chick stomach development and differentiation. (A) Whole-mount in situ hybridization of E6 uninfected (control) and *GFP*-expressing gastrointestinal tracts using *SM22*, *BAPX1* and *SOX10* riboprobes. Scale bars, 1 mm. (B) Transverse sections of E7 control and *GFP*-expressing stomachs analysed either by in situ hybridization using the *LIX1* riboprobe or by immunofluorescence with anti-αSMA and anti-CALPONIN antibodies. Nuclei were visualized with Hoechst. (C) Serial transverse sections of E7 control and *GFP-*expressing stomachs analysed by immunofluorescence using anti-PH3 antibodies. Nuclei were visualized with Hoechst. Graph represents the quantification of PH3-positive cells. ns, Not significant. (JPG 1115 kb) Additional file 11: Table S2. Gene-specific chick primers used for RT-qPCR. List of primer sequences used for transcript amplification by RT-PCR. (PDF 36 kb) Additional file 12. Supporting data. (XLSX 46 kb)
