# Supplementary figures and images for: LIX1 regulates YAP1 activity and controls the proliferation and differentiation of stomach mesenchymal progenitors
Source: BMC Biol. 2016 Apr 28;14:34. doi: 10.1186/s12915-016-0257-2 (PMC4848777; doi:10.1186/s12915-016-0257-2)

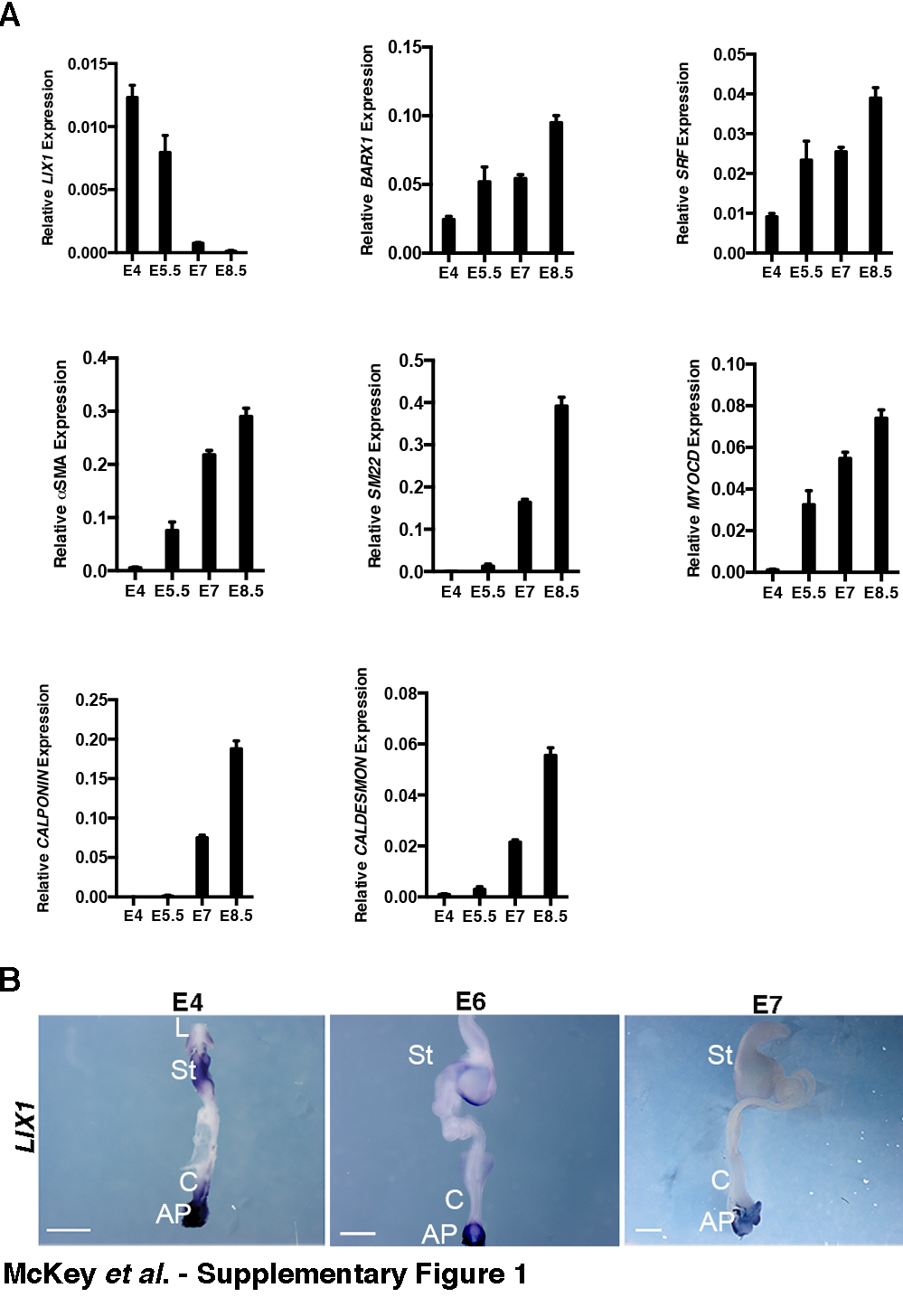

Supplement: Additional file 1: Figure S1. — Comparison of LIX1 expression with smooth muscle cell development markers. (A) RT- qPCR analysis of the endogenous relative mRNA expression of LIX1, BARX1, SRF, aSMA, SM22, MYOCARDIN (MYOCD), CALPONIN and CALDESMON expression in E4, E5.5, E7 and E8.5 stomachs. (B) LIX1 whole-mount in situ hybridization of E4 to E7 gastrointestinal tracts. Strong LIX1 expression is detected in the stomach and colon as early as E4, decreasing over time to become no longer detectable from E7 onwards. We also noted a strong expression of LIX1 in the associated lungs and anal plate. Scale bars, 1 mm. L, Lung; St, Stomach; C, Colon; AP, Anal plate. (JPG 427 kb) [file 12915_2016_257_MOESM1_ESM.jpg]

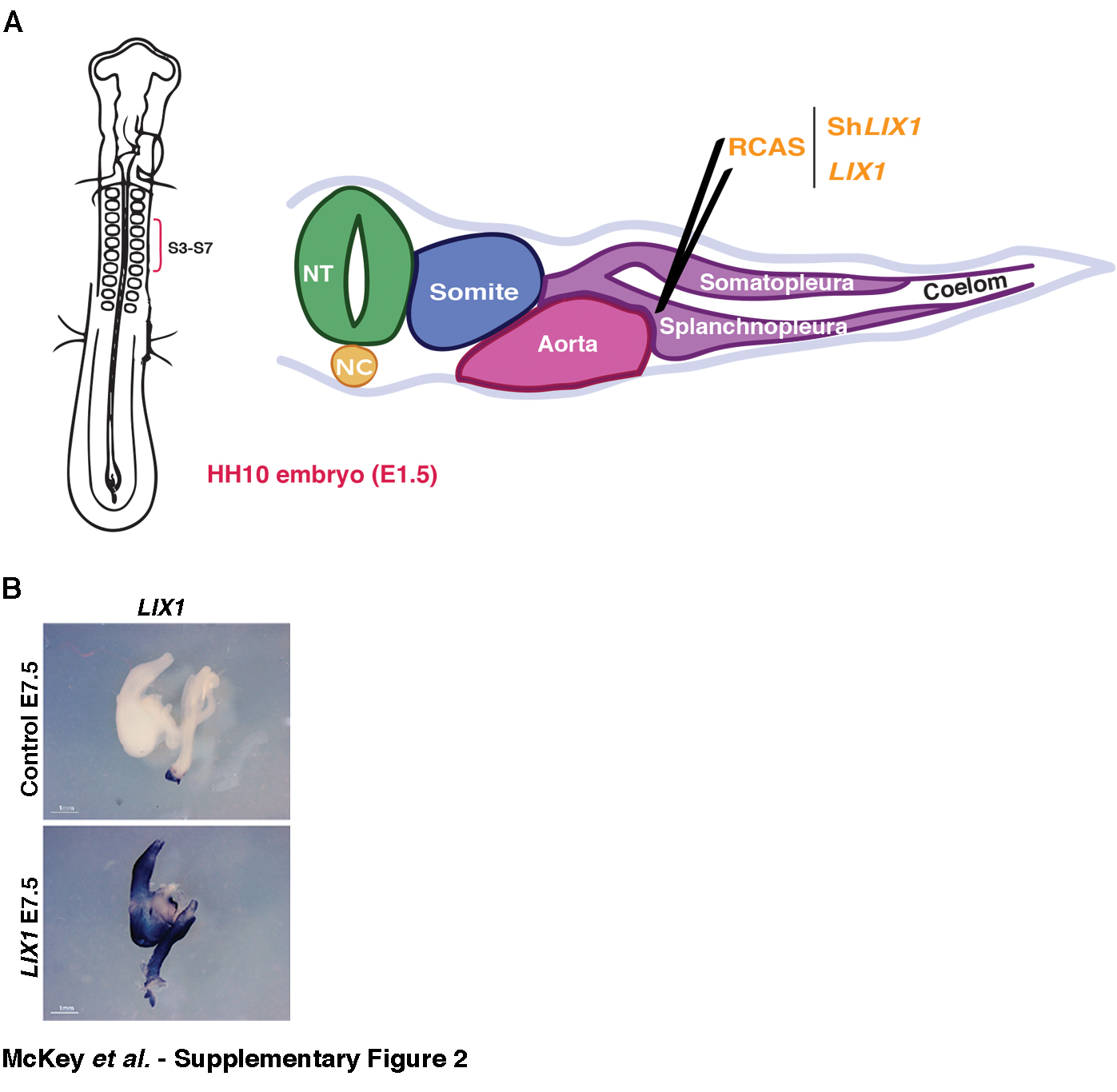

Supplement: Additional file 2: Figure S2. — RCAS retroviral infection. (A) Schematic representation of RCAS retroviral infection. RCAS(A)-ShLIX1 or RCAS(B)-LIX1 retroviral particles are injected in the splanchnopleura, between somites 3 and 7 of embryos at E1.5. NT, Neural tube; NC, Notochord. (B) LIX1 whole-mount in situ hybridization on E7.5 control or LIX1-expressing stomachs. (JPG 460 kb) [file 12915_2016_257_MOESM2_ESM.jpg]

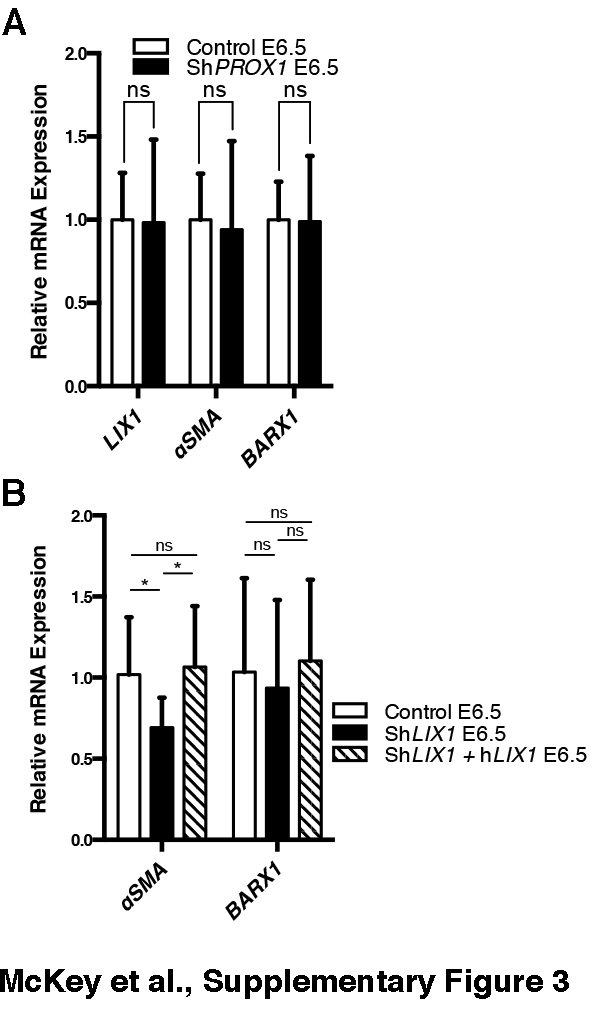

Supplement: Additional file 3: Figure S3. — Specificity of the ShLIX1 construct for LIX1 mRNA. (A) Analysis of LIX1, αSMA and BARX1 expression by RT-qPCR in E6.5 control and RCAS-ShPROX1-expressing stomachs. Data were normalized to GAPDH expression. Normalized expression levels were converted to fold changes. Values are presented as the mean ± standard error of the mean of n = 8 controls vs. n = 8 ShPROX1-expressing stomachs. ns, Not significant by one-tailed (for LIX1) or two-tailed (for αSMA and BARX1) Mann–Whitney tests. (B) Analysis of LIX1, αSMA and BARX1 expression by RT-qPCR in E6.5 control stomachs, RCAS(A)-ShLIX1- expressing stomachs or RCAS(A)-ShLIX1/RCAS(B)-hLIX1-co-expressing stomachs. Data were normalized to GAPDH expression. Normalized expression levels were converted to fold changes. Values are presented as the mean ± standard deviation of n = 12 controls vs. n = 8 ShLIX1 vs. n = 8 ShLIX1+ hLIX1-expressing stomachs. *P < 0.05 by one-tailed Mann–Whitney test. ns, Not significant. (JPG 170 kb) [file 12915_2016_257_MOESM3_ESM.jpg]

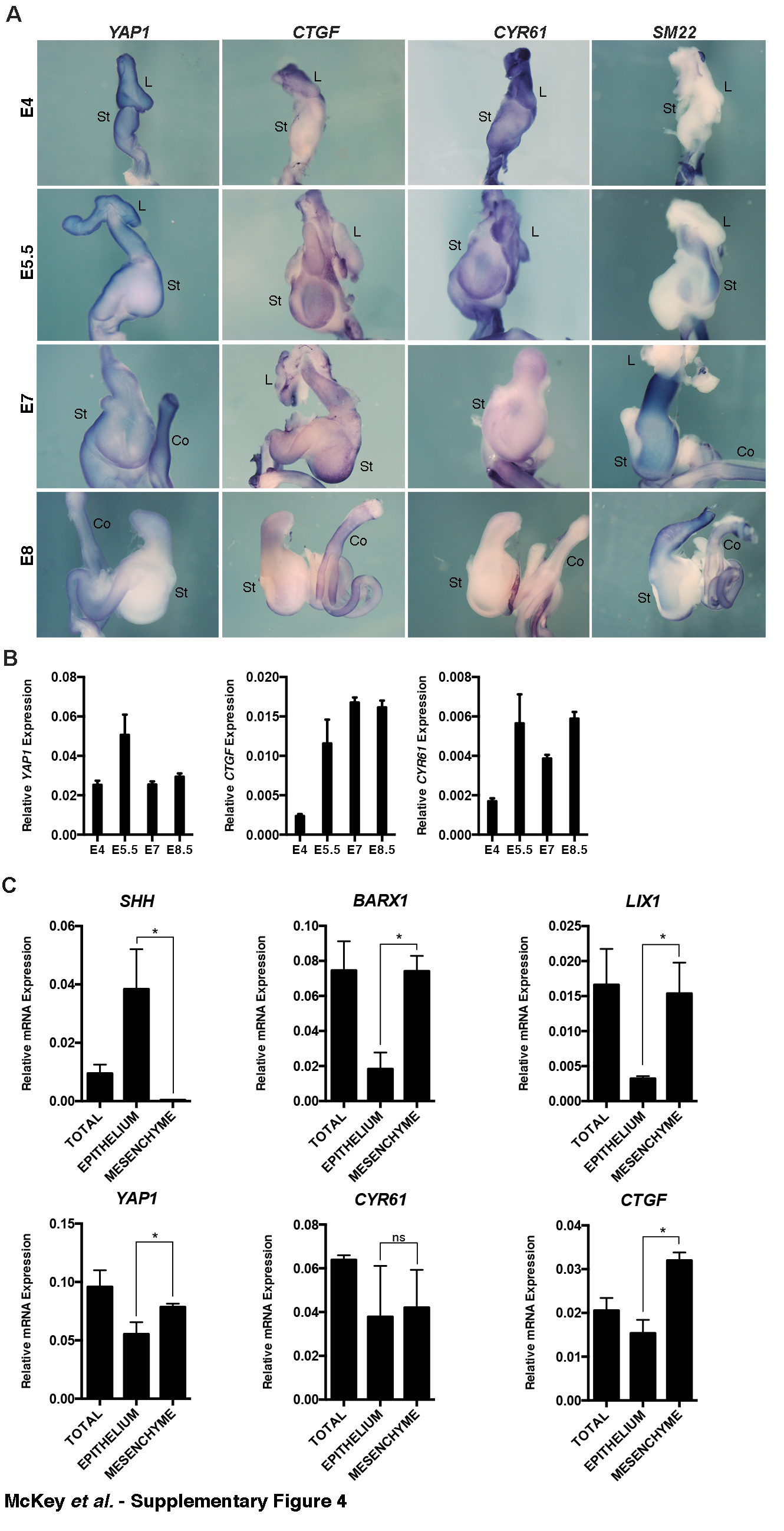

Supplement: Additional file 5: Figure S4. — Expression pattern of YAP1 and its transcriptional target genes during gastrointestinal development. (A) YAP1, CTGF, CYR61 and SM22 whole-mount in situ hybridization of E4–8 gastrointestinal tracts. L, Lung; St, Stomach; Co, Colon. (B) RT- qPCR analysis of the endogenous relative mRNA expression of YAP1, CTGF, CYR61 in E4–8.5 stomachs. (C) RT-qPCR analysis of the endogenous relative mRNA expression of YAP1, CYR61, CTGF, SHH, αSMA and BARX1 in mesenchymal and endodermal layers dissected from E5 stomachs. Total, Whole stomach; Epithelium, Epithelial layer; Mesenchyme, Mesenchymal layer. Values are presented as the mean ± standard deviation of n = 3 experiments. *P < 0.05 by one-tailed Mann–Whitney test. ns, Not significant. Raw data for panel C are shown in Additional file 12. (JPG 1455 kb) [file 12915_2016_257_MOESM5_ESM.jpg]

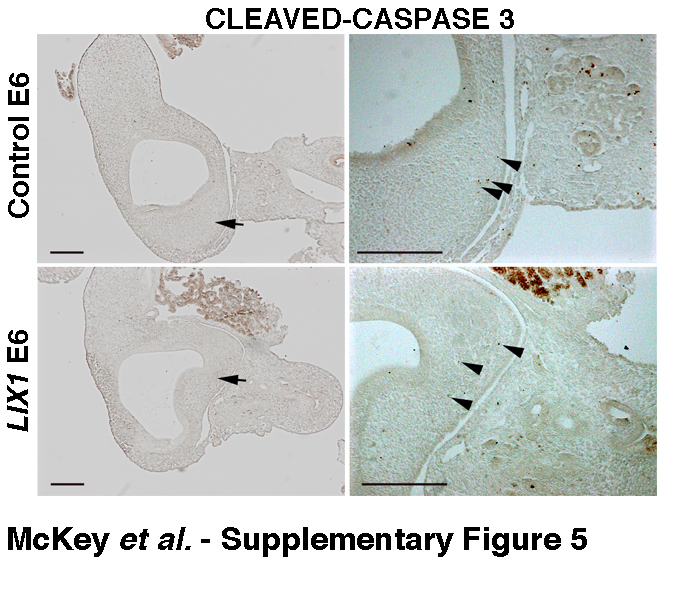

Supplement: Additional file 6: Figure S5. — Sustained LIX1 expression does not affect apoptosis. Transverse sections of E6 control and LIX1-expressing stomachs analysed by immunohistochemistry with anti-cleaved CASPASE-3 antibodies. Black arrows indicate the area imaged at high power in the cleaved-CASPASE-3 panels. Black arrowheads point to cleaved CASPASE-3-positive apoptotic cells. (JPG 402 kb) [file 12915_2016_257_MOESM6_ESM.jpg]

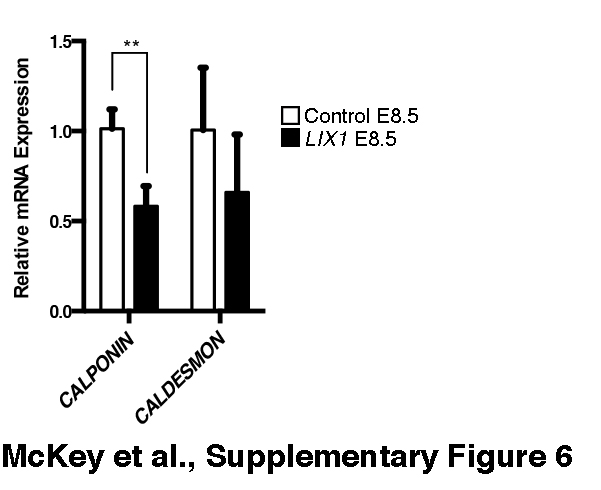

Supplement: Additional file 7: Figure S6. — Sustained LIX1 expression hinders smooth muscle cell differentiation. Analysis of CALPONIN and CALDESMON expression by RT-qPCR in E8.5 control GFP- and LIX1-expressing stomachs. Data were normalized to GAPDH expression. Normalized expression levels were converted to fold changes. Values are presented as the mean ± standard deviation of n = 4 controls vs. n = 5 LIX1-expressing stomachs. **P < 0.01 by one-tailed Mann–Whitney test. ns, Not significant. Raw data are shown in Additional file 12. (JPG 91 kb) [file 12915_2016_257_MOESM7_ESM.jpg]

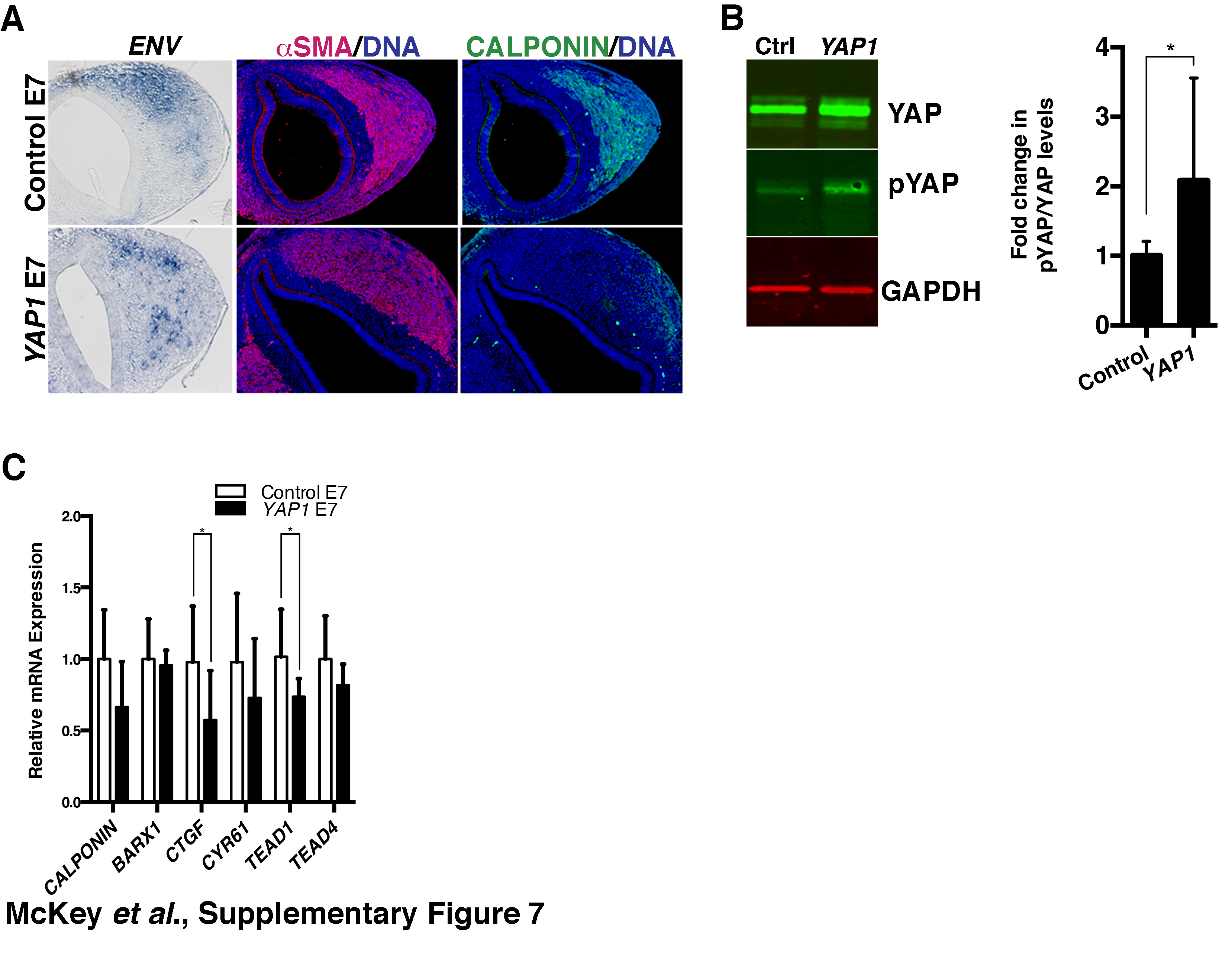

Supplement: Additional file 8: Figure S7. — Sustained YAP1 expression hinders smooth muscle cell differentiation. (A) Serial transverse sections of E7 control GFP- and YAP1-expressing stomachs analysed either by in situ hybridization using the ENV riboprobe or by immunofluorescence with anti-αSMA and anti-CALPONIN antibodies. Nuclei were visualized with Hoechst. (B) Western blot analysis of YAP and phospho-YAP (72 kDa) levels in protein extracts from control GFP- and YAP1-expressing stomachs. Equal loading was verified by GAPDH expression (37 kDa). Graph represents the quantification of western blot data. Normalized expression levels were converted to fold changes. Values are presented as the mean ± standard deviation of n = 6 controls vs. n = 6 YAP1-expressing stomachs. *P < 0.05 by one-tailed Mann–Whitney test. (C) RT-qPCR analysis of relative mRNA expression in E7 control GFP- and YAP1-expressing stomachs. Data were normalized to GAPDH expression. Normalized expression levels were converted to fold changes. Values are presented as the mean ± standard deviation of n = 8 controls vs. n = 6 YAP1-expressing stomachs. *P < 0.05 by one-tailed Mann–Whitney test. (JPG 2004 kb) [file 12915_2016_257_MOESM8_ESM.jpg]

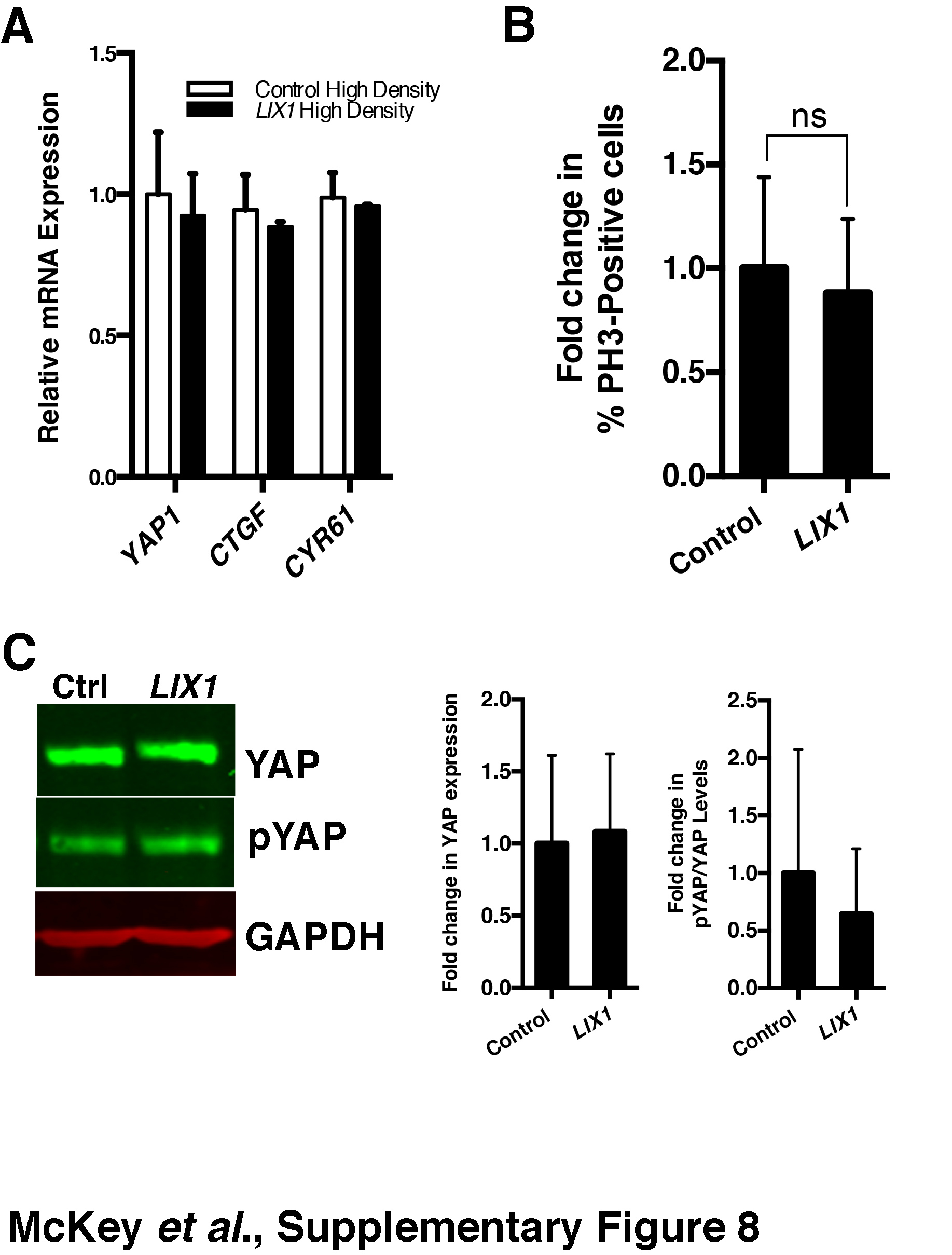

Supplement: Additional file 9: Figure S8. — The pro-proliferative effect of LIX1 is abolished when DF-1 cells are seeded at high density. (A) RT-qPCR analysis of relative mRNA expression in DF-1 cells infected with RCAS(B)-GFP control or RCAS(B)-LIX1 plated at high density and harvested at day 1. Data were normalized to GAPDH expression. Normalized expression levels were converted to fold changes. Values are presented as the mean ± standard deviation (SD) of n = 2 experiments. Raw data for panel A are shown in Additional file 12. (B) Examination of proliferation in GFP-expressing cells (control) and LIX1-expressing cells. Graphs represent the quantification of PH3-positive cells. Normalized expression levels were converted to fold changes. Values are presented as the mean ± SD of n = 7 experiments. ns, Not significant by two-tailed Mann–Whitney test. (C) Western blot analysis of YAP and phospho-YAP (72 kDa) levels in protein extracts from GFP- and LIX1-expressing cells. Equal loading was verified by GAPDH expression (37 kDa). Graph represents the quantification of western blot data. Normalized expression levels were converted to fold changes. Values are presented as the mean ± SD of n = 7. (JPG 721 kb) [file 12915_2016_257_MOESM9_ESM.jpg]

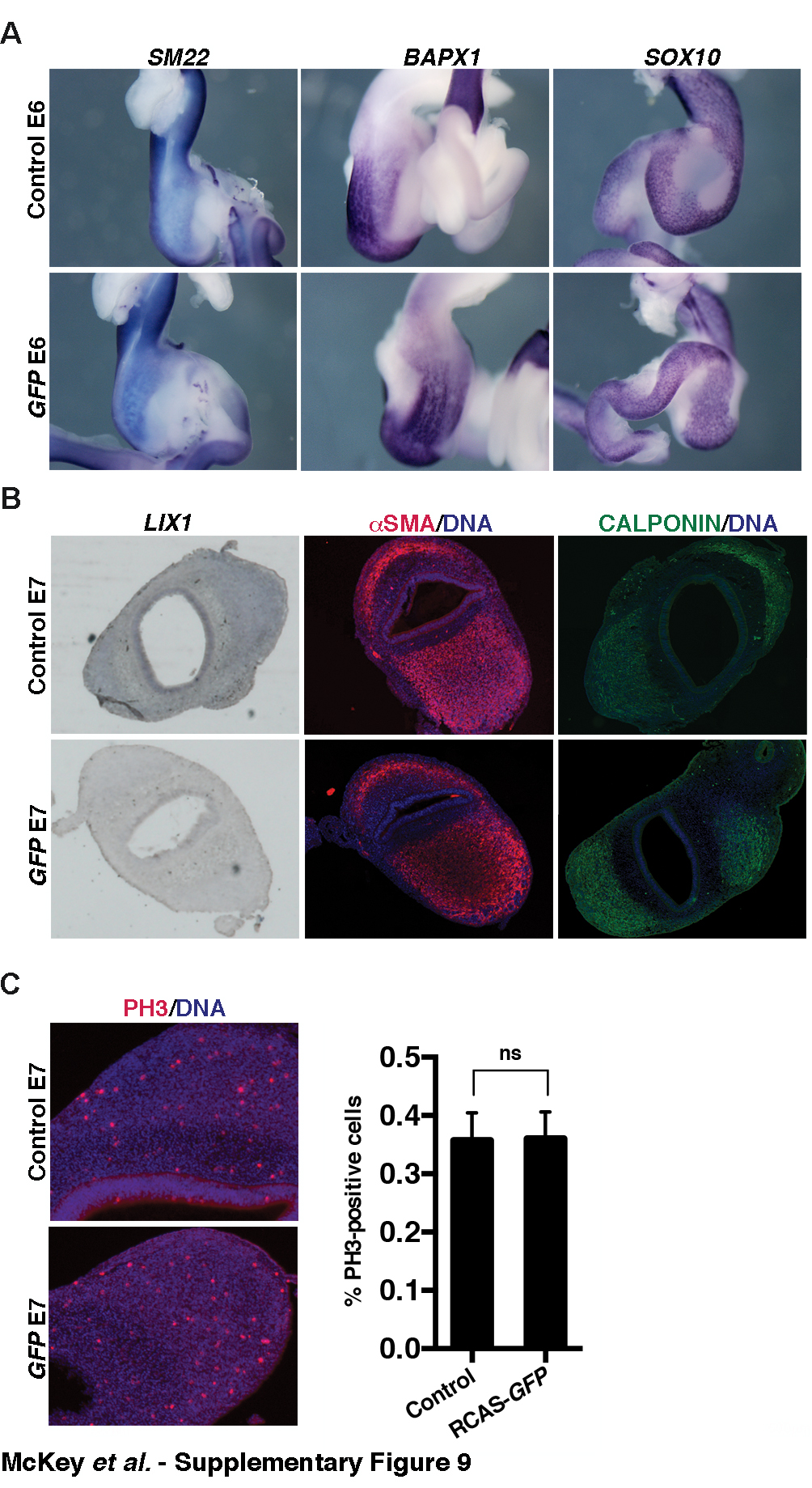

Supplement: Additional file 10: Figure S9. — Infection with RCAS-GFP retroviruses does not affect chick stomach development and differentiation. (A) Whole-mount in situ hybridization of E6 uninfected (control) and GFP-expressing gastrointestinal tracts using SM22, BAPX1 and SOX10 riboprobes. Scale bars, 1 mm. (B) Transverse sections of E7 control and GFP-expressing stomachs analysed either by in situ hybridization using the LIX1 riboprobe or by immunofluorescence with anti-αSMA and anti-CALPONIN antibodies. Nuclei were visualized with Hoechst. (C) Serial transverse sections of E7 control and GFP-expressing stomachs analysed by immunofluorescence using anti-PH3 antibodies. Nuclei were visualized with Hoechst. Graph represents the quantification of PH3-positive cells. ns, Not significant. (JPG 1115 kb) [file 12915_2016_257_MOESM10_ESM.jpg]
