# Supplementary material for: LIX1 regulates YAP1 activity and controls the proliferation and differentiation of stomach mesenchymal progenitors
Source: BMC Biol. 2016 Apr 28;14:34. doi: 10.1186/s12915-016-0257-2 (PMC4848777; doi:10.1186/s12915-016-0257-2)
Supplement: Additional file 4: Table S1. — Phenotype quantification. Quantification of embryos harbouring an abnormal stomach muscle phenotype, as demonstrated by in situ hybridization or immunostaining, following injection of RCAS(A)-ShLIX1 (LIX1 loss-of-function) or RCAS(B)-LIX1 (LIX1 gain-of-function). (PDF 40 kb) [file 12915_2016_257_MOESM4_ESM.pdf]

Table S1 Phenotype quantification

Experiments	Phenotypes	Embryos analysed	Embryos with phenotype	Phenotype %
***LIX1* loss-of-function**				
E4.5 *LIX1* ISH	Down-regulation	36	22	61
E6.5 SM22 ISH	Smaller domain	23	14	60.87
E7 PH3 IS	Less PH3-positive cells	12	11	91.67
***LIX1* gain-of-function**				
E4.5 *SM22* ISH	Early expression	16	11	68.75
E6.5 *SM22* ISH	Expanded domain	30	17	57
E6.5 *BAPX1* ISH	Expanded domain	13	9	69
E6.5 *SOX10* ISH	Ectopic staining	20	12	60
E6 PH3 IS	More PH3-positive cells	15	15	100
E7 PH3 IS	Less PH3-positive cells	16	16	100

ISH, in situ hybridization ; IS, immunostaining.
