# Supplementary material for: LIX1 regulates YAP1 activity and controls the proliferation and differentiation of stomach mesenchymal progenitors
Source: BMC Biol. 2016 Apr 28;14:34. doi: 10.1186/s12915-016-0257-2 (PMC4848777; doi:10.1186/s12915-016-0257-2)
Supplement: Additional file 11: Table S2. — Gene-specific chick primers used for RT-qPCR. List of primer sequences used for transcript amplification by RT-PCR. (PDF 36 kb) [file 12915_2016_257_MOESM11_ESM.pdf]

Table S2 Gene-specific chick primers used for RT-qPCR.

Targets	Forward primer (5'-3')	Reverse primer (5'-3')	Amplicon (bp)
αSMA	CTG TAT GCT TCT GGG CG	GCA GTG GTC ACA AAG GAG	188
BARX1	CCG CTA CCG CAG TTT CA	GCT CCG CCT TCA GAA CG	152
CALDESMON	GAA ACA CGC CAA GGA CG	CTT CCA GCA TAG CAT TTG TAT CATC	258
CALPONIN	GAC GAA GGG CAA CAA CG	CAA GGG CTG GTC TGT CC	202
CTGF	GGA GAA GCA GAG CAG ACT	GCA GAC ACC ACA GAA CTT AG	172
CYR61	TGA TTG CCA TCG TGA AGG G	GGG ATT GTC GTT GGT GAC T	167
GAPDH	CGT CCT CTC TGG CAA AG	TCA CGC TCC TGG AAG ATA G	177
LIX1	ATT GTC TTT CAG CTT CTG CAT	TGC TCC TTC ATA ATC CAG TCC	152
MYOCD	CTT CTG TCA GCA ACA CCC	AAG ACT GCG ACT GGT AAC	300
SM22	TGA GCA GGG ATG TCC AGT	AGC CAA TGA TGT TCT TGC C	500
SRF	CAG GCA CCA CAG CAA AC	CAG CCG AGA CTG GGA TT	328
TEAD1	CTCAGGCTGGTGGAGTT	GTCGCTGTAAGAATGGTTTGC	108
TEAD4	ACCCTATGCTCTACAGCC	CAACCCTTCGTCCTTGC	101
UBIQUITIN	GGG ATG CAG ATC TTC GTG AAA	CTT GCC AGC AAA GAT CAA CCT T	147
YAP1	CCA GAG ATG AAA GCA CAG ATA G	GCT TCA AGG TAG TCT GGG AAT	163
